# Degree of improving TMS focality through a geometrically stable solution of an inverse TMS problem

**DOI:** 10.1016/j.neuroimage.2021.118437

**Published:** 2021-07-28

**Authors:** S.N. Makarov, W.A. Wartman, G.M. Noetscher, K. Fujimoto, T. Zaidi, E.H. Burnham, M. Daneshzand, A. Nummenmaa

**Affiliations:** a Electrical and Computer Engineering Department, Worcester Polytechnic Institute, Worcester, MA 01609 USA; b Center for Devices and Radiological Health (CDRH), FDA, Silver Spring, MD 20993 USA; c Athinoula A. Martinos Center for Biomedical Imaging, Massachusetts General Hospital, Harvard Medical School, Boston, MA 02115 USA

## Abstract

The Transcranial Magnetic Stimulation (TMS) inverse problem (TMS-IP) investigated in this study aims to focus the TMS induced electric field close to a specified target point defined on the gray matter interface in the M 1 _HAND_ area while otherwise minimizing it. The goal of the study is to numerically evaluate the degree of improvement of the TMS-IP solutions relative to the well-known sulcus-aligned mapping (a projection approach with the 90° local sulcal angle). In total, 1536 individual TMS-IP solutions have been analyzed for multiple target points and multiple subjects using the boundary element fast multipole method (BEM-FMM) as the forward solver.

Our results show that the optimal TMS inverse-problem solutions improve the focality – reduce the size of the field “hot spot ” and its deviation from the target – by approximately 21–33% on average for all considered subjects, all observation points, two distinct coil types, two segmentation types, two intracortical observation surfaces under study, and three tested values of the field threshold. The inverse-problem solutions with the maximized focality simultaneously improve the TMS mapping resolution (differentiation between neighbor targets separated by approximately 10 mm) although this improvement is quite modest.

Coil position/orientation and conductivity uncertainties have been included into consideration as the corresponding de-focalization factors. The present results will change when the levels of uncertainties change. Our results also indicate that the accuracy of the head segmentation critically influences the expected TMS-IP performance.

## 1. Introduction

Transcranial Magnetic Stimulation (TMS) activation zones are linked to the induced electric field in the cortex. The activation zone can be anywhere where the field exceeds the activation threshold, which does vary over space. Furthermore, different directions of the induced electric field (or electric current) within these domains can excite different intracortical circuits or the same circuits but at different sites (Di Lazzaro et al., 2013, Di Lazzaro et al., 2008, Bashir et al., 2013, Raffin et al., 2015).

The domains of field maxima with respect to a certain field component – total, tangential, or normal to cortical surfaces – are determined by the coil winding geometry, coil position and orientation in space, head geometry including the unique gyral pattern of the subject as well as tissue properties. Several such domains may simultaneously be generated, e.g., a primary domain in the originally targeted motor cortex or M1 and secondary “hot spots” in the somatosensory and/or premotor cortexes. Also, the domain size(s) and type of the dominant field component may vary.

All three electric field components – total, tangential, and normal – separately, are considered in different TMS models of activation mechanisms for different neural populations (Weise et al., 2020, Balderston et al., 2020, Dubbioso et al., 2017, Reijonen et al., 2019, Bungert et al., 2017, Aberra et al., 2020, Fox et al., 2004, Krieg et al., 2013, Miranda et al., 2007, Salvador et al., 2011, Laakso et al., 2018). Most important is likely the magnitude of the total TMS electric field along the gyral crown (Weise et al., 2020, Bungert et al., 2017, Aberra et al., 2020), although other studies have observed pre-dominantly sulcal activations (Fox et al., 2004, Krieg et al., 2013, Miranda et al., 2007, Salvador et al., 2011, Laakso et al., 2018). Detailed neuronal models have elucidated the different neuronal elements and their activation thresholds (Aberra et al., 2020). From the macroanatomical perspective, the activated region is considered as a comprise of the locations where the field intensity is highest (Weise et al., 2020).

A number of computational approaches have been developed to solve the TMS “forward problem ”: determine the corresponding intracranial E-field distributions given the coil position/orientation and the subject-specific anatomically realistic volume conductor geometry via a numerical solution of the quasistatic Maxwell equations using either the finite element method (cf. Laakso and Hirata, 2012, Saturnino et al., 2019, Gomez et al., 2020) or, more recently, also the boundary element method (cf. Stenroos and Koponen, 2019, Makarov et al., 2018, Makarov et al., 2020).

On the other hand, the TMS “inverse problem ” or TMS-IP studied in this paper aims to determine the coil position and orientation that will optimally focus the total TMS induced electric field around a user-specified cortical target point and/or domain and minimize it otherwise.

In this study, the performance or degree of improvement of a TMS-IP solution will be quantified as compared to the well-known sulcus-aligned mapping of the M1_HAND_ (the hand knob Yousry et al., 1997) area. The sulcus-aligned mapping is a common projection approach for the primary motor cortex or M1 with the 90° local sulcal angle (Raffin et al., 2015, Dubbioso et al., 2017, Reijonen et al., 2019), also called $\text{CURVED}-90_{flex}^{\circ }$ (Raffin et al., 2015). This promising approach allows one to readily probe the within-hand motor somatotopy (Bashir et al., 2013, Raffin et al., 2015) in M1_HAND_ with neuronavigated TMS that follows the sulcal shape and creates a tissue field/current perpendicular to the central sulcus at all mapping sites (Raffin et al., 2015). Furthermore, using sulcus-shape based mapping, novel evidence has been provided that fast sensorimotor integration in M1_HAND_ displays a center-surround organization, engaging center inhibition and surrounding facilitation (Dubbioso et al., 2017).

In other words, we aim to answer the following questions:

- By how much would the TMS-IP solution help us to increase the focality of the $\text{CURVED}-90_{flex}^{\circ }$ mapping; and
- By how much would it help us to better differentiate between neighbor targets in M1_HAND_ ?

For a comprehensive and statistically accurate characterization of the TMS-IP performance, we analyze 16 subject-specific head models, each with six target points in the M1_HAND_ area separated as described in Refs. Raffin et al., 2015, Dubbioso et al., 2017. We further employ two different coil types (a large MRiB91 coil and a small CoolB35 coil, both of MagVenture), two different intracortical field observation surfaces, and two different segmentation models for the same subject (mri2mesh and headreco), both implemented within the SimNIBS segmentation pipeline (Saturnino et al., 2019).

As a focality metric or the cost function of the TMS-IP solution, we choose an average absolute deviation (AAD) of the field magnitude, ||***E***||, from the target point with respect to a certain field percentile. Three values of the field threshold – 70, 80 and 90% – are investigated in this study. The total electric field is evaluated either on the mid-surface between gray and white matter or on a surface shifted toward the white matter boundary, with the separation ratio of 4:1. While the mid-surface approximately corresponds to the cortical layer 2/3 (L2/3), the second observation surface approximately corresponds to the (bottom of) cortical layer 5 (L5, cf., for example, Aberra et al., 2020).

The major stability considerations for the TMS-IP solution to be taken into account are

- Inverse-problem solution stability and deviation with respect to geometrical uncertainties in the relative coil-head position and with respect to all three coil coordinates and three independent orientation angles;
- Inverse-problem solution stability and deviation caused by variations in tissue properties;
- Inverse-problem solution stability and deviation with respect to the segmentation accuracy.

The numerical implementation of the TMS-IP solution uses the boundary element fast multipole method (BEM-FMM) (Makarov et al., 2018, Makarov et al., 2020) as the forward solver and a simple gradient descent search method with a small step size in a full six-dimensional search space as the inverse solver. This search space includes three independent coil coordinates and three independent coil rotation angles as suggested in the recent TMS protocol (Balderston et al., 2020). The expected outcome is the best position and orientation of the coil for the given subject head model and for the given target point.

The gradient search method is tested and confirmed against the most reliable exhaustive approach applied previously in a comprehensive and detailed relevant study (Weise et al., 2020). The method can be applied for studying multiple geometrical model variations and arbitrary field observation surfaces, arbitrary coil positions and orientations different from those strictly tangential to the scalp, and it does not require extra mesh conditioning.

The main finding of this study shows that the TMS-IP solutions may further improve the focality of the sulcus-aligned motor mapping approach for the total electric field in the gyral crown. This relative improvement is stable, consistent with an average value around 21–33%, and is weakly dependent on the coil type and the observation surface type. However, it is moderate. Coil position/orientation and conductivity uncertainties have been included into consideration as the corresponding de-focalization factors.

Next, it is demonstrated that the TMS-IP solution might somewhat improve the “somatotopic resolution” of TMS for the M1_HAND_ area of the precentral gyrus (the hand knob) which could in principle be “scanned” with a relatively high resolution when moving the focal hot spot for the likely most important magnitude of the TMS total field (Weise et al., 2020, Bungert et al., 2017, Aberra et al., 2020) along the gyral crown. Additionally, improvements in the deviation of the absolute field maximum from target and other parameters are quantified. All these results are critically dependent on the segmentation model used because different segmentation models lead to quite different electric field distributions (Puonti et al., 2020).

The article is organized as follows. Section 2 Materials and Methods describes the major parameters of the present TMS-IP solution, including subject models, segmentation models, focality metrics, as well as numerical implementation and its validations. Sections 3, 4, 5 Results describe all obtained TMS-IP solutions, including individual results, averaged data, target-wise data, and threshold-wise data. Section 6 Discussion lists and discusses major features of the obtained TMS-IP solution step by step, attempts to link our modeling results to the recently obtained experimental data (Weise et al., 2020, Raffin et al., 2015), and finally discusses the major limitations of the study. Section 7 concludes the article.

## 2. Materials and Methods

Major parameters of the present inverse-problem solution are initially summarized in Table 1 and then described in detail below in this section. 16 × 2 × 2 × 6 × 3 = 1152 individual inverse problem solutions have been analyzed following different parameter permutations from Table 1.

To check overall robustness of the mid-surface representation, the inverse problem has additionally been solved for another observation surface that is shifted toward white matter (4:1, ∼Layer 5). Those computations have been performed for the 80% field threshold only. This resulted in an extra set of 16 × 2 × 2 × 6 × 1 = 384 TMS IP solutions.

### 2.1. Subject MRI data

MRI T1/T2 data for sixteen Human Connectome Project (HCP) healthy subjects (Van Essen et al., 2012, head only) with an initial isotropic voxel resolution of 0.7 mm have been selected (Htet et al., 2019). The subjects IDs are listed in Table 1 above. The reason for selecting those subjects was a sufficiently good skull quality observed after segmentation with the mri2mesh option of SimNIBS (Htet et al., 2019). The skull quality was inspected visually and the sixteen models without visible dents/holes had been selected.

### 2.2. Segmentation and meshing of MRI data

**Fig. 1.**
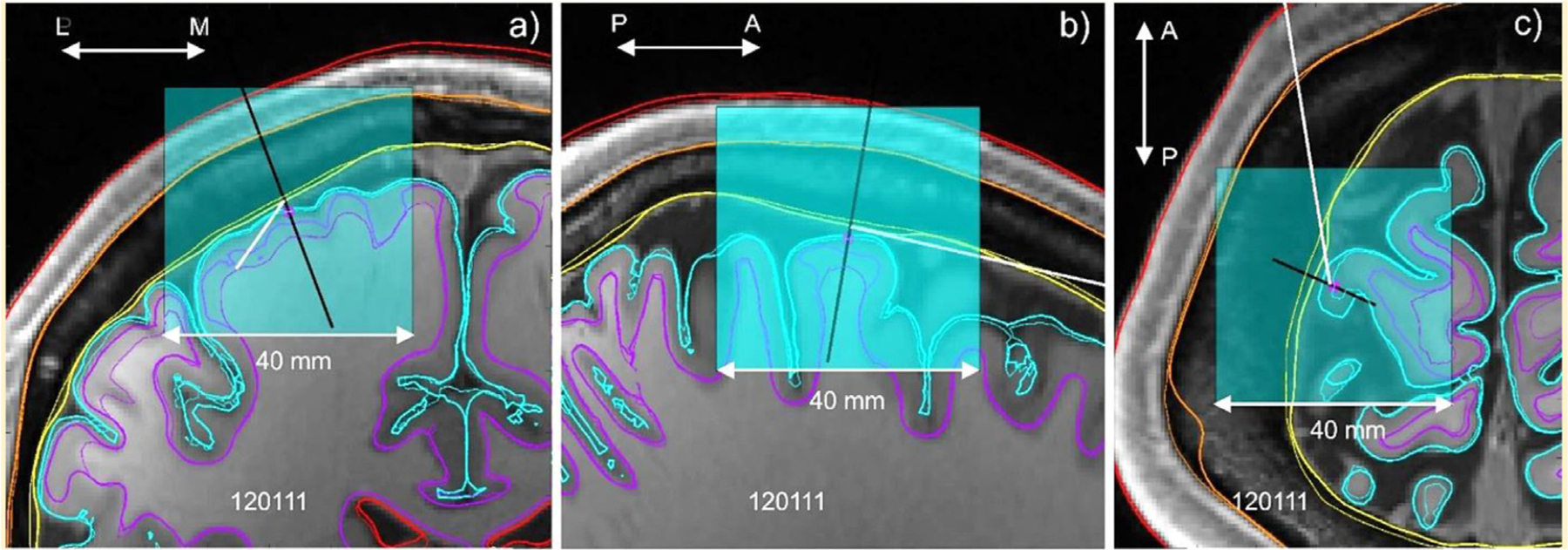
Segmentation data for mri2mesh (thick curves) and headreco (thin curves) superimposed onto T1 data for Connectome subject 120111 in three principal planes (coronal, sagittal, transverse) when targeting the motor hand area, M1_HAND_. The target point is a small magenta circle; the coil axis is a black line; the coil handle direction is a white line drawn in (c).

Two types of the automated segmentation routines available in SimNIBS 3.1 software have been used: mri2mesh (based on FreeSurfer Fischl (2012) and FSL) and headreco (based on SPM/CAT Structural Brain Mapping Group (2021)), both with the default options. By using the two segmentation routines, we aim to evaluate the effect of intra-segmentation variability on the computed TMS focality. It is known from the literature that the segmentation results appear some-what different in both cases (Seiger et al., 2018), (Righart et al., 2017). As an example, Fig. 1 shows both segmentation types for Connectome subject 120111 from Table 1 targeting the motor hand area, M1_HAND_, of the left hemisphere and superimposed onto the original T1 images.

Both segmentation routines create triangular surface meshes for six main brain compartments under study: skin, skull, cerebrospinal fluid (CSF), gray matter, white matter, and ventricles. The cerebellum was combined with the white matter in either case. Every subject dataset was thus segmented twice, which resulted in 32 computational models in total. The typical model size is approximately 1 M (million) triangular facets in either case.

Table 2 quantifies the resulting average distance, *d*, between CSF shells, gray matter shells, and white matter shells for both segmentation routines used in this study. This distance was determined as a mean of the shortest distances from every triangle centroid of one shell to all triangle centroids of the other shell, whose triangular mesh was previously refined with the ratio of 1:36 to achieve a good accuracy. These results are only valid for the superior part of the cerebral cortex, strictly above the corpus callosum. Deviations in the M1_HAND_ area do exceed the mean values.

### 2.3. Selection of TMS coils

**Fig. 2.**
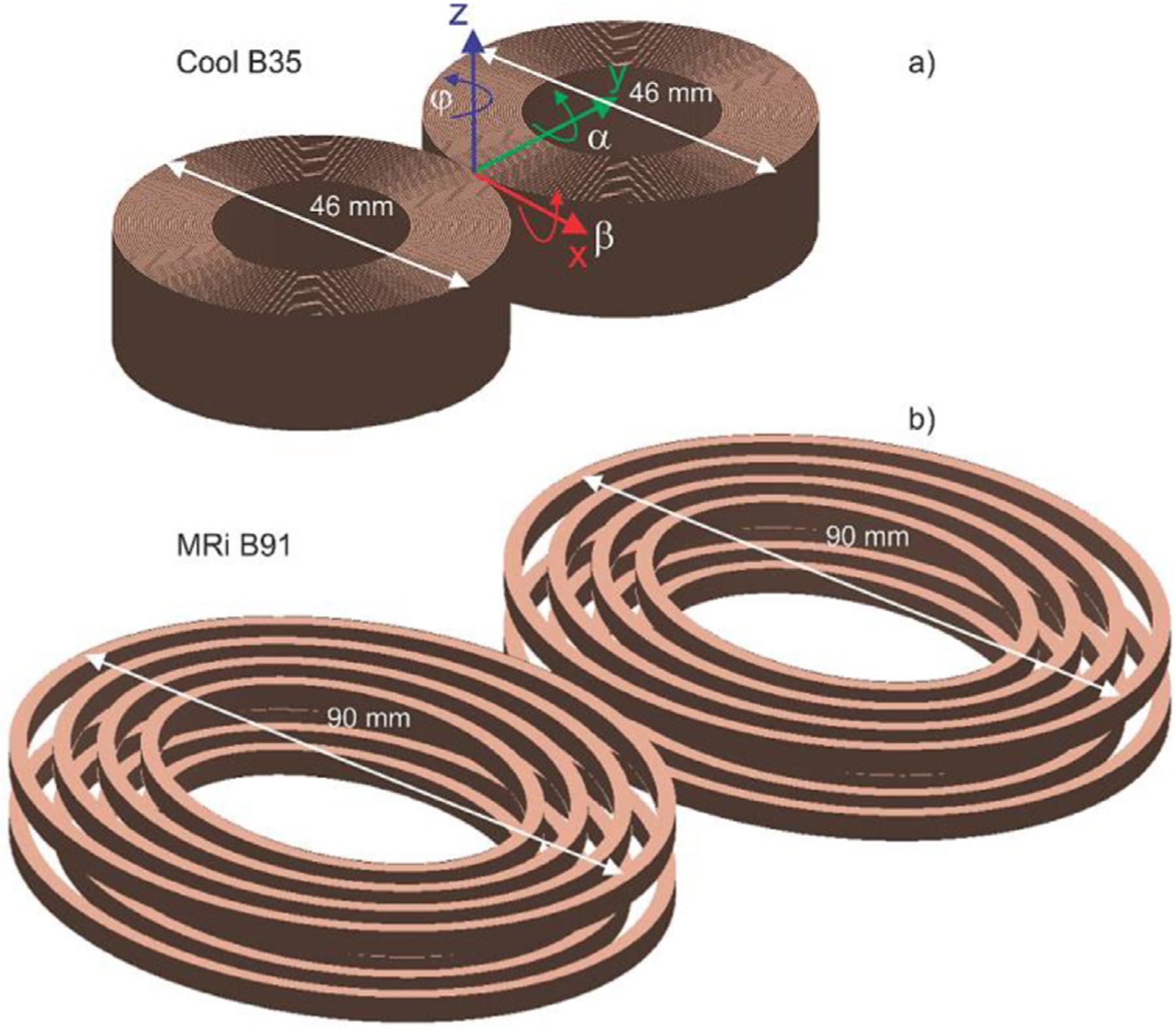
Two coil types (Cool B35) and MRiB91 used in this study to scale. The coil dimensions in the direction of the coil handle (direction of the induced electric field) differ by a factor of approximately 2. Principal rotation angles of the coil – pitch *α*, roll *β*, and yaw *φ*– are shown in Fig. 2a.

A small CoolB35 TMS coil (Fig. 2, top) and a relatively large elliptical MRiB91 TMS coil (Fig. 2, bottom), both of MagVenture, Inc., have been converted to CAD models using the manufacturer’s datasheets and then used in this study. By using two different coils with the size ratio of approximately two, we aim to evaluate the effect of the absolute coil size on the computed TMS focality. The coils are approximated by ∼100,000–120,000 elementary current elements (with the skin depth effect included) and the coil fields are computed via the fast multipole method (Makarov et al., 2019).

### 2.4. Selection of TMS target domains and target points

**Fig. 3.**
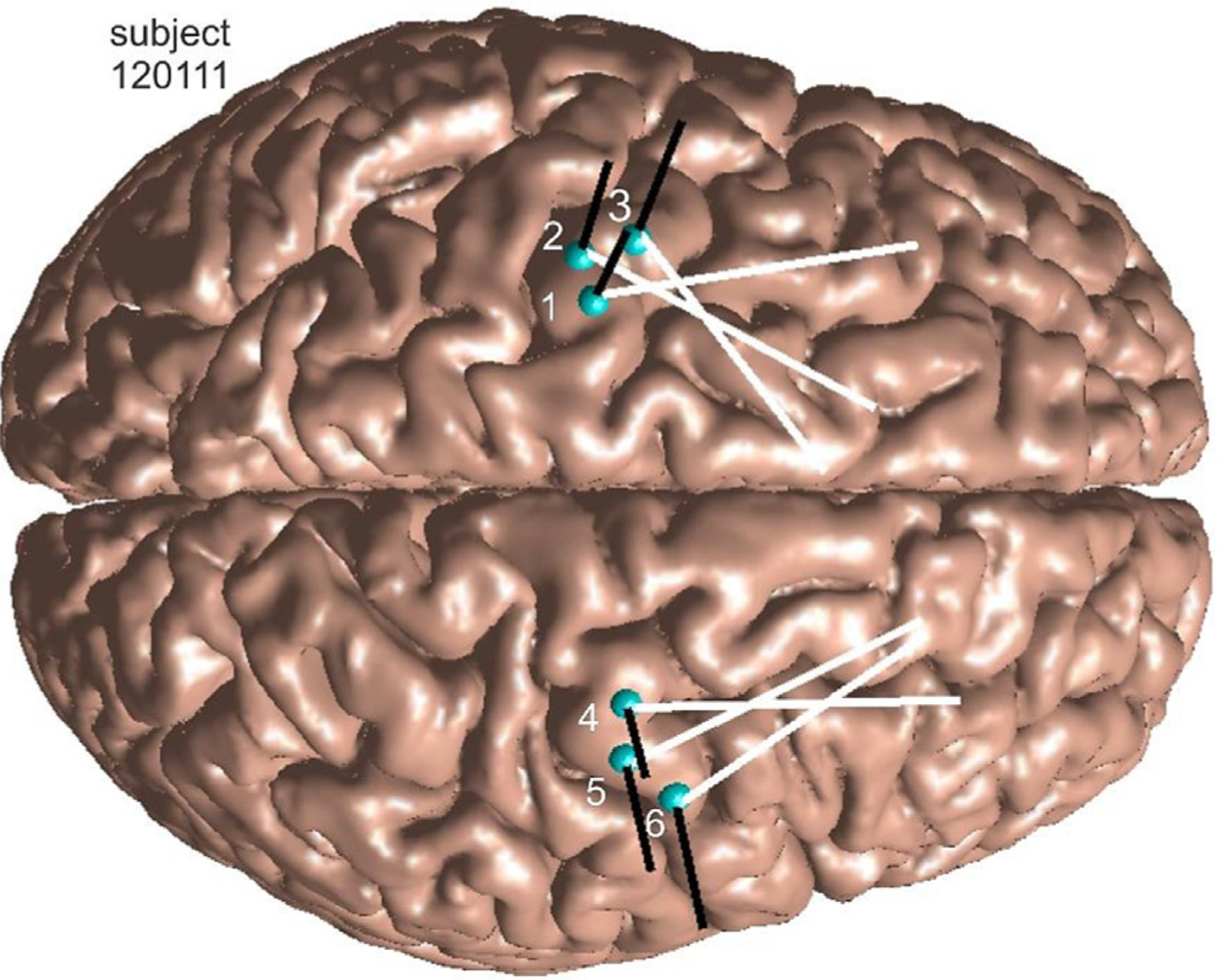
Six TMS target points ***T*** within left and right M1_HAND_ areas of subject 120111 (superimposed onto mri2mesh gray matter segmentation) as indicated by blues spheres. Directions of the coil axis (black lines) and the directions of the induced electric field (coil handle, white lines) are shown, which are used in the sulcus-aligned motor mapping.

For every subject, six TMS target points, ***T***, within the M1_HAND_ areas on the crown of the precentral gyrus have been identified on the gray matter interface in global MRI coordinates – three points per every hemisphere. These points are illustrated in Fig. 3 for subject 120111 by blue spheres. The M 1 _HAND_ areas were defined with the help of a neuroanatomist. Exactly the same target points (16 × 6 = 96 in total) have been used for both segmentation types (mri2mesh and headreco). Following the relevant experimental setup in (Raffin et al., 2015, Dubbioso et al., 2017), the average linear distance between the target points was maintained at 10 mm, with the standard deviation of less than 1.2 mm. The center of a target cloud approximately coincides with the center of the respective M1_HAND_ area found using its posterior convexity as central reference (Yousry et al., 1997).

Simultaneously, Fig. 3 shows the directions of the coil axis (black lines) and the directions of the induced electric field or current (the coil handle, white lines) which will be used in the sulcus-aligned motor mapping – the initial guess of an optimization solution.

### 2.5. Selection of initial coil positions: sulcus-aligned coil mapping

The initial coil position is based on a direct projection onto the cortical surface with the field direction being perpendicular to the nearest sulcal wall. It is constructed by the following three steps:

- make the coil centerline passing through a given target point ***T*** defined in global MRI coordinates on the well-defined gray matter interface;
- make the coil centerline perpendicular to the skin surface at the skin-centerline intersection, and position the coil bottom at the distance of 10 ± 0.25 mm from the skin surface (to account for the coil case and hair thickness in an average sense) along this centerline;
- make the dominant coil field direction (coil handle) approximately perpendicular to the sulcal wall (of the central sulcus for M1_HAND_) nearest to the target point, which is the sulcus-aligned approach (Raffin et al., 2015, Dubbioso et al., 2017, Reijonen et al., 2019).

The examples of the initial coil positioning for subject 120111 are shown in Fig. 3.

### 2.6. Selection of observation domains and field type

To be consistent with the previous relevant studies (Weise et al., 2020, Saturnino et al., 2019) and with the biophysical activation mechanisms, the first *E*-field observation domain was chosen as a mid-surface between gray and white matter. The total electric field (field magnitude, ||***E***|| was sampled on this surface. Another observation surface was also chosen, shifted closer toward the white matter boundary, with the separation ratio of 4:1. While the mid surface approximately corresponds to the cortical layer 2/3 (L2/3), the second observation surface approximately corresponds to the (bottom of) cortical layer 5 (L5, cf., for example, Aberra et al., 2020).

The two observation surfaces were created by finding the shortest vector distances from every triangle centroid of the gray matter shell to all triangle centroids of the white matter shell, whose triangular mesh was subdivided (using the barycentric subdivision) with the ratio of 1:36 to achieve a sufficient accuracy. Then, the nodes of the observation surfaces were defined as the centroids of gray matter facets moved along the established vector distances, with the appropriate relative separation from both boundaries (1:1 or 4:1).

### 2.7. Cost function (focality metric) of the inverse problem and its physical interpretation

Consider any target point ***T*** on the gray matter interface of a subject’s head model shown, for example, in Fig. 3. The goal of the present TMS-IP is to vary TMS coil position and orientation in order to maximize the field on the observation surface close to the target point ***T*** and minimize it everywhere else. In other words, the best coil focality is sought in the vicinity of the target point. Other formulations of the inverse problem are indeed possible.

The search space is a six-dimensional space (denoted by ℜ^6^) that includes three Cartesian coordinates of the coil center and three coil rotation angles (pitch-roll-yaw) shown in Fig. 2. The only physical constraint imposed on the search space implies that the *nearest* distance from any part of the coil metal conductor (without the plastic case) to the skin of the subject under study is no less than 10 mm. This constraint is enforced by monitoring the minimum distance between the scalp and the coil windings, for every coil position and orientation. If this distance appears less than 10 mm, the corresponding case is automatically excluded from the set of tested cases.

The cost function to be minimized is defined in this study as the mean or average absolute deviation (*AAD*) of the field maxima from the target point ***T*** in 3D. Formally,

(1) $$AAD=mean\left(\left\|T-T_{i}\right\|\right)[\text{mm}],\;i=1,\ldots ,N$$

with ||⋅|| being the Euclidian norm, and ***T***_*i*_ being *N* nodes of the observation surface where the electric field magnitude values ||***E***|| are greater than or equal to a certain percentage of the *known* absolute maximum of the electric field, for example 70, 80, or 90% of 100 V/m.

The measure of Eq. (1) can therefore be interpreted as an effective radius of a focal domain centered at the target where the field values are greater than or equal to the prescribed value. It does not square the distance from the target, so it is less affected by a few extreme observations than the variance and standard deviation. Similar non-squared measures are used in robust data analysis (Ding et al., 2006). Threshold values of 70, 80, or 90% have been investigated in this study; an interpolation could be made between the respective results to estimate the effect of intermediate values.

The threshold-based cost function in Eq. (1) is meant to support motor mapping techniques, which are based on individual motor threshold measurements and which predict a certain percentage of the maximum stimulator output and an estimated electric-field strength e.g., 80 V/m (cf., for example, Julkunen et al., 2009, Awiszus, 2003). When the problem geometry, pulse strength, and the target position are all given, the TMS-IP solution based on Eq. (1) will vary the coil position and orientation and predict:

- The smallest effective radius of the focal domain where the field does exceed a certain percentage of the absolute field maximum, for example 80%.
- The absolute field maximum itself, for example 100 V/m.

Within this focal domain, the field will thus exceed 100 × 0.8 = 80 V/m. If a different value of the field maximum will be obtained, the stimulator output needs to be adjusted using linear scaling.

For subthreshold TMS, other non-threshold based definitions may perhaps be more appropriate such as a geodesic distance to all other nodes, weighted by their field magnitudes.

### 2.8. Target differentiation or “somatotopy” metric

In addition to the absolute focality, it is useful to know how different the field would become at the immediate neighbor target points, after the *AAD* has been minimized for one of them. Note that in the present case and in the background experimental studies (Raffin et al., 2015, Dubbioso et al., 2017, Weise et al., 2020), the neighboring target points are separated by approximately 10 mm (7–10 mm in Weise et al., 2020). The “somatotopy” metric may thus be defined as an average relative differentiation (*ARD*) of the electric field values ***E_T_*** and $T_{T_{n}}$ between the selected target point ***T***, and its immediate neighboring target points ***T***_*n*_, respectively. One has

(2) $$ARD=1-mean\left(\frac{\left\|E_{T_{n}}\right\|}{\left\|E_{T}\right\|}\right)[a.u.],\;n=1,2$$

In the present case, the number of immediate neighbors, *n*, in Eq. (2) is equal to two. Eq. (2) predicts a zero *ARD* when the fields at the neighbor targets are equal to the field at the selected target. On the other hand, it predicts the *ARD* of one (or 100%) when the fields at the neighbor targets are vanishingly small compared to the field at the target in question (the maximum differentiation). Note that every field value in Eq. (2) is in fact an average field for four nodes of the mid-surface or the 4:1 cortical surface, which are closest to the target in question. This averaging domain size is slightly less than 3 mm in the present case and for the given segmentation resolution.

The metric given by Eq. (2) does not depend on the specific value of the field threshold used. Therefore, it does not change for the sulcus-aligned mapping with different field thresholds. However, since the cost function and the TMS-IP solution both depend on the field threshold, it will slightly vary for TMS-IP solutions with different field thresholds.

### 2.9. Gradient descent search method: test against exhaustive search. Forward solver

A simple (perhaps the simplest) gradient descent search method with a sequential variable-by-variable update and a relatively small step size was considered as a prospective candidate. For sixteen test cases considered (target points #1 and #2 were tested for two subjects, for two coil types, and two observation surfaces), the gradient descent search method generated nearly the same results as the straightforward exhaustive search method. In one situation where this was not the case, the exhaustive-search solution appeared *unstable* and had to be reverted to the former solution as explained later.

Therefore, the gradient descent method in the six-dimensional space of three coil coordinates and three rotation angles shown in Fig. 1a was chosen in this study, along with a relatively small and fixed step size. The constraint on the minimum coil-scalp distance of 10 mm discussed previously was additionally imposed. The method is based on variations

(3) $$\begin{array}{l} x=x_{0}\pm \Delta x,\;y=y_{0}\pm \Delta y,\;z=z_{0}\pm \Delta z,\;\alpha =\alpha_{0}\pm \Delta \alpha ,\\ \beta =\beta_{0}\pm \Delta \beta ,\;\varphi =\varphi_{0}\pm \Delta \varphi \end{array}$$

executed and processed sequentially. First, the coil azimuthal angle *φ* = *φ*_0_ ± Δ*φ* in Fig. 2 is varied about its initial state *φ*_0_, and the angle providing the minimum of the cost function is selected. Next, angles *α* = *α*_0_ ± Δ*α* and *β* = *β*_0_ ± Δ*β*, which characterize the direction of the coil axis, are varied in a similar fashion. Finally, we vary three spatial coordinates of the coil: *x* = *x*_0_ ± Δ*x, y* = *y*_0_ ± Δ*y, z* = *z*_0_ ± Δ*z* sequentially and then update the cost function (and coil position) at every individual iteration step.

The iterative process repeats itself until the solution saturates (i.e., no longer changes or converges). Given sufficiently small step values, no step refinement was required. We found that the values: Δ*x* = Δ*y* = Δ*z* = 2 mm and Δ*φ* = Δ*α* = Δ*β* = 0.1 *rad* = 5.7° provide convergence in 4–10 iterations. A typical *AAD* convergence (the first target point of the first subject model, MRiB91 coil, headreco segmentation) is: 7.8, 7.4, 6.8, 6.1, 5.8, 5.6, 5.5, 5.5 mm (stop, converged).

For the forward-problem solution, we use the boundary element fast multipole method formulated in terms of induced surface charge density *ρ*(***r***) residing at the conductivity interfaces or BEM-FMM (Makarov et al., 2018, Makarov et al., 2020, GitHub Repository 2021). The method possesses high numerical accuracy, which was shown to exceed that of the comparable finite element method of the first order (Gomez et al., 2020). Most recent efforts made it possible to obtain the forward-problem solution in approximately 12–14 s for a head model with 0.9 million facets and with 7 brain compartments using a 2.8 GHz workstation. The RAM requirements (6–12 Gbytes) of the forward solver are moderate; the number of cores is more critical: 16 or more cores are preferred. Without parallelization of the search algorithm, a single-target optimization run (the individual TMS-IP solution) requires approximately 3–5 min to complete.

### 2.10. Test of self-convergence

While the BEM-FMM forward solver itself was tested previously (Makarov et al., 2018, Makarov et al., 2020, Gomez et al., 2020), the inverse-problem solution was not. To do so, we have rerun

- 96 inverse problems with a different number of iterative passes (12 instead of 6);
- 8 inverse problems with a finer mesh resolution (using a 4 × barycentric mesh refinement).

In every case, the relative differences in the mean *AAD* values reported below in the following section did not exceed 1–5%, which was considered satisfactory.

### 2.11. Solution stability vs geometrical uncertainties in coil position. Stability correction

The stability of the inverse problem solution against uncertainties in coil position and orientation, as well as model segmentation defects is critical. The geometrical stability has been checked for every single TMS-IP solution by introducing a relative average de-focalization, *DF*, in the following way. Let’s assume that in Eq. (3), index 0 now denotes the final solution with the cost function *AAD*_*final*_. Its variations corresponding to Δ*x* = Δ*y* = Δ*z* = 1.5 mm and Δ*φ* = Δ*α* = Δ*β* = 0.1 *rad* = 5.7° have been tested. These dimensions approximately correspond to the accuracy of a modern robotized TMS navigator or cobot (Axilum Robotics 2021) (controlled 2 mm linear and 4° angular coil placement accuracy). These solution variations give us 12 more *AAD* values. The relative average defocalization *DF*_*coil*_ (always greater or equal than one) is found in the form:

(4) $$DF_{coil}=\underset{n=0:12}{mean}\left(AAD_{n}\right)/AAD_{final}\geq 1$$

When *DF* exceeded 1.25 at the last iteration, the inverse-problem solution was classified as unstable. This has been found in approximately only 1–2% of all considered cases. The iterative solution was then reverted to the second to last iteration and the stability check was performed again, etc., until the inequality *DF <* 1.25 was met. We were able to accomplish this task in all the considered cases.

### 2.12. TMS-IP solution result. Measure of success

Below, index *initial* will indicate the initial guess – the sulcus-aligned mapping. Index *final* will indicate the final TMS-IP solution. These indexes may relate to *AAD* from Eq. (1), to *ARD* from Eq. (2), and to defocalization *DF*_*coil*_ from Eq. (4). After the TMS-IP solution is obtained, the cost function for the initial coil position, *AAD*_*initial*_, is compared with the final cost function, *AAD*_*final*_. The dimensionless relative difference of these or the absolute difference (in mm) is the field focality improvement. If either difference is substantial, the usefulness of the TMS-IP solution will be proven.

Both *AAD*_*initial*_ and *AAD*_*final*_ are modeling estimates. They should further be adjusted to include the effect of defocalization, *DF*_*coil*_, from Eq. (4), the effect of inaccuracy due to tissue conductivity variations, and the effect of segmentation inaccuracy to obtain a more realistic estimate. While the two last mechanisms will likely contribute equally and will thus cancel out in the relative difference, the coil position/orientation-based inaccuracy clearly contributes more substantially (it will be proven later) when a higher focality is sought by tweaking the coil position/orientation itself. Therefore, the corrected relative focality improvement to within the main order of magnitude could be formulated in the following form:

(5) $$Focality\;Improvement=1-\frac{DF_{coil}^{final}\times AAD_{final}}{DF_{coil}^{initial}\times AAD_{initial}}$$

If the numerator and denominator on the right-hand side of Eq. (5) coincide, the focality improvement is 0%. If the numerator is close to zero, the focality improvement would approach 100%.

Along with the relative estimate of Eq. (5), absolute estimates are worth visualizing as given below in Figs. 10 and 11. For that purpose, one needs other defocalization expressions explicitly. As to the conductivity uncertainties, we will employ here the results of previous very comprehensive relevant studies reported in (Saturnino et al., 2019) and (Weise et al., 2020), respectively. There and for TMS, the mean of the total electric field on the gyral crown is characterized by a relatively small error of generally less than 5% for a wide range of electrical conductivity values assigned to the different tissue types (Weise et al., 2020). We thereforeassume the corresponding defocalization factor in the form

(6) $$DF_{cond}=1.05$$

## 3. Subject - and location-wise non-averaged results

### 3.1. Field distributions before (sulcus-aligned mapping) and after (TMS-IP solution) optimization at the mid-surface

**Fig. 4.**
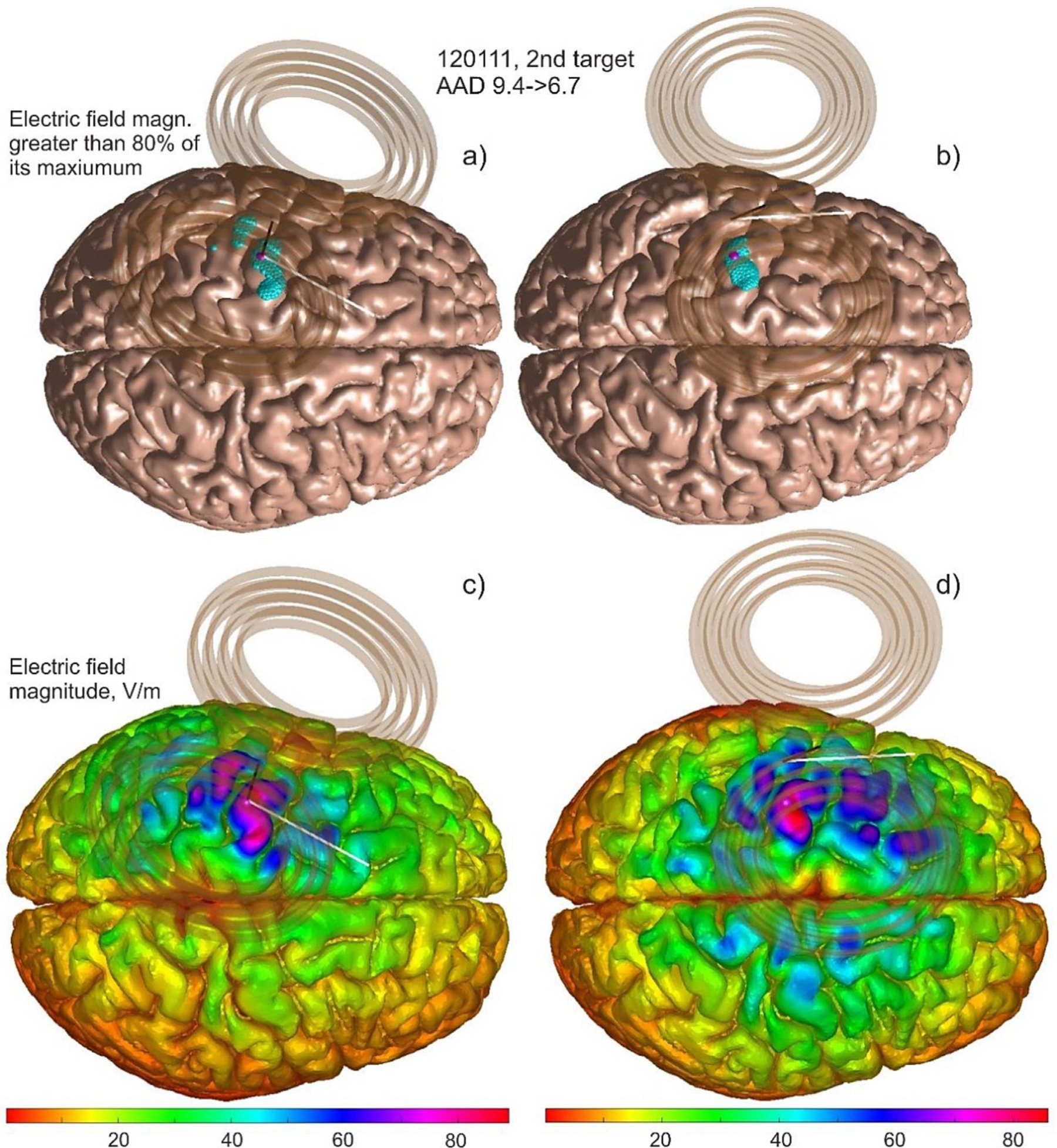
MRiB91 Large coil of MagVenture targets the left motor hand area of Connectome subject 120111; TMS pulse strength is 9.4e7 A/s. Target point ***T***_2_ (M1_HAND_) on the gray matter interface is shown by a small magenta sphere. a) and c) – 80^th^ percentile and continuous electric field magnitude, respectively, for the sulcus-aligned mapping; b) and d) – the same result after the *AAD* optimization. For plotting, the total electric field at the mid-surface is projected on the gray matter interface and then displayed in both binary and continuous forms.

Fig. 4 demonstrates a typical optimization result in subplots b) and d) as compared to the original sulcus-aligned mapping shown in a) and c) for Connectome subject 120111 and for the large MRiB91 coil. Here, we use an 80% field threshold in the cost function versus the (optimized) maximum field. The total field at the mid-surface is projected on the gray matter interface and then plotted, both in binary (a, b) and continuous (c, d) form.

In Fig. 4, the *AAD* value (which could be treated as an “effective radius” of the suprathreshold area) only moderately decreases from 9.4 mm to 6.7 mm. This is at the expense of increasing the overall subthreshold field spread, mostly in the premotor area. At the same time, the initially observed second suprathreshold maximum in the somatosensory area presumably becomes subthreshold and is thus eliminated from consideration.

**Fig. 5.**
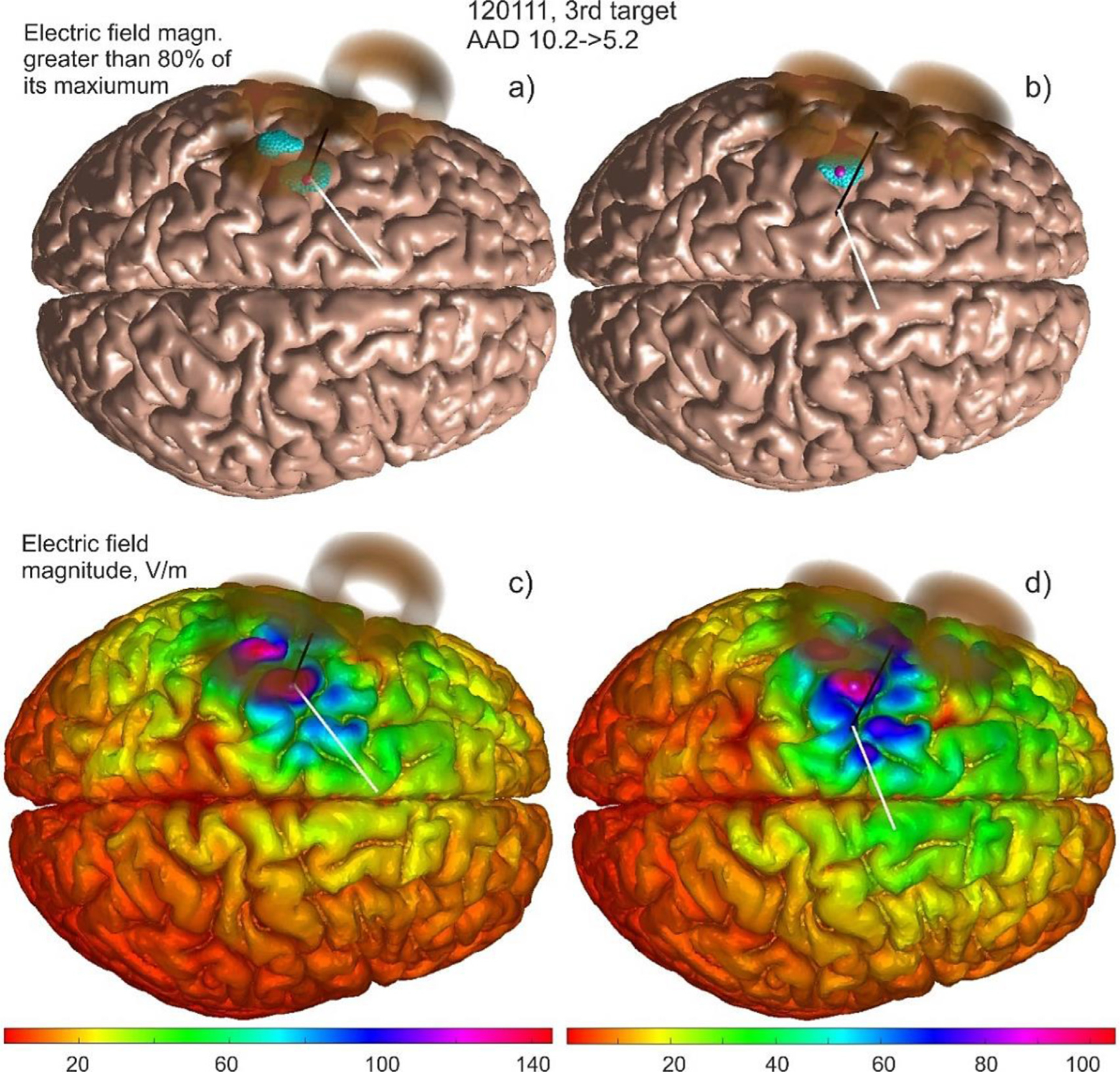
Small CoolB35 coil of MagVenture targets left motor hand area of Connectome subject 120111; TMS pulse strength is 9.4e7 A/s. Target point ***T***_3_ (M1_HAND_) on the gray matter interface is shown by a small magenta sphere. a) and c) – 80^th^ percentile and continuous electric field magnitude, respectively, for the sulcus-aligned mapping; b) and d) – the same result after the *AAD* optimization. For plotting, the total electric field at the mid-surface is projected on the gray matter interface and then displayed in both binary and continuous forms.

Next, Fig. 5 demonstrates a typical optimization result in subplots and d) as compared to the original sulcus-aligned mapping in a) and for the same Connectome subject 120111, but for the small CoolB35 coil and for a different target point. The total field at the mid-surface is again projected on the gray matter interface and then plotted, both in binary (a, b) and continuous (c, d) form.

In Fig. 5, the *AAD* value (the “effective radius” of the suprathreshold area) now decreases more significantly as compared to the previous case, namely from 10.2 mm to 5.2 mm. This is again at the expense of increasing the subthreshold field spread, in the premotor area as well as in the M1_HAND_ itself. At the same time, the initially observed second suprathreshold maximum in the somatosensory area again becomes subthreshold. Similar results have been observed for the 4:1 observation surface in both cases.

The larger spread of a weaker field over a wider area in Figs. 4 and 5 seems to be the unavoidable physical cost of a TMS-IP solution. We could unfortunately do nothing about it since it is likely a physical property of the existing coil geometries and coil fields. Better coils (coils with magnetic cores, or coil arrays, or etc.) might perhaps resolve this issue in future.

### 3.2. Typical optimization results for the mid-surface

**Fig. 6.**
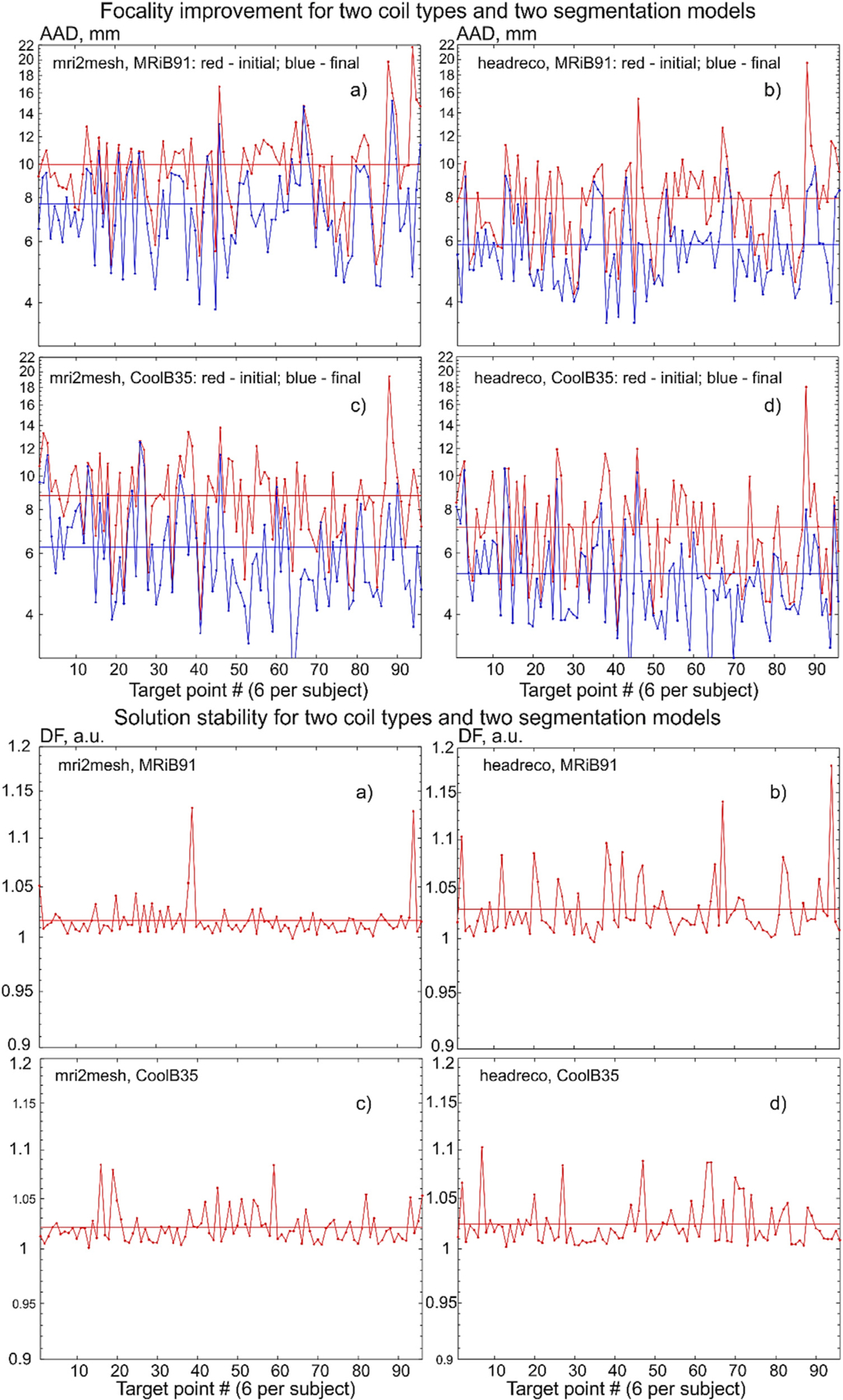
Target-by-target *AAD* and defocalization *DF*_*coil*_ results for the mid-surface at the 80% field threshold. Target points are numbered sequentially. Averaged data are shown by straight lines.

The corresponding target-by-target individual raw data for all 16 subjects and all target points are illustrated in Fig. 6 below when the cost function with the field threshold of 80% is used. Namely, Fig. 6 shows target-by-target *AAD* in mm and dimensionless defocalization *DF*_*coil*_ results for the mid-surface for both segmentation models and for the two coil types. The target points are numbered sequentially within each subject model as shown in Fig. 3 so that the argument in Fig. 6 runs from 1 to 96.

### 3.3. Typical optimization results for the 4:1 intracortical observation surface between gray and white matter

**Fig. 7.**
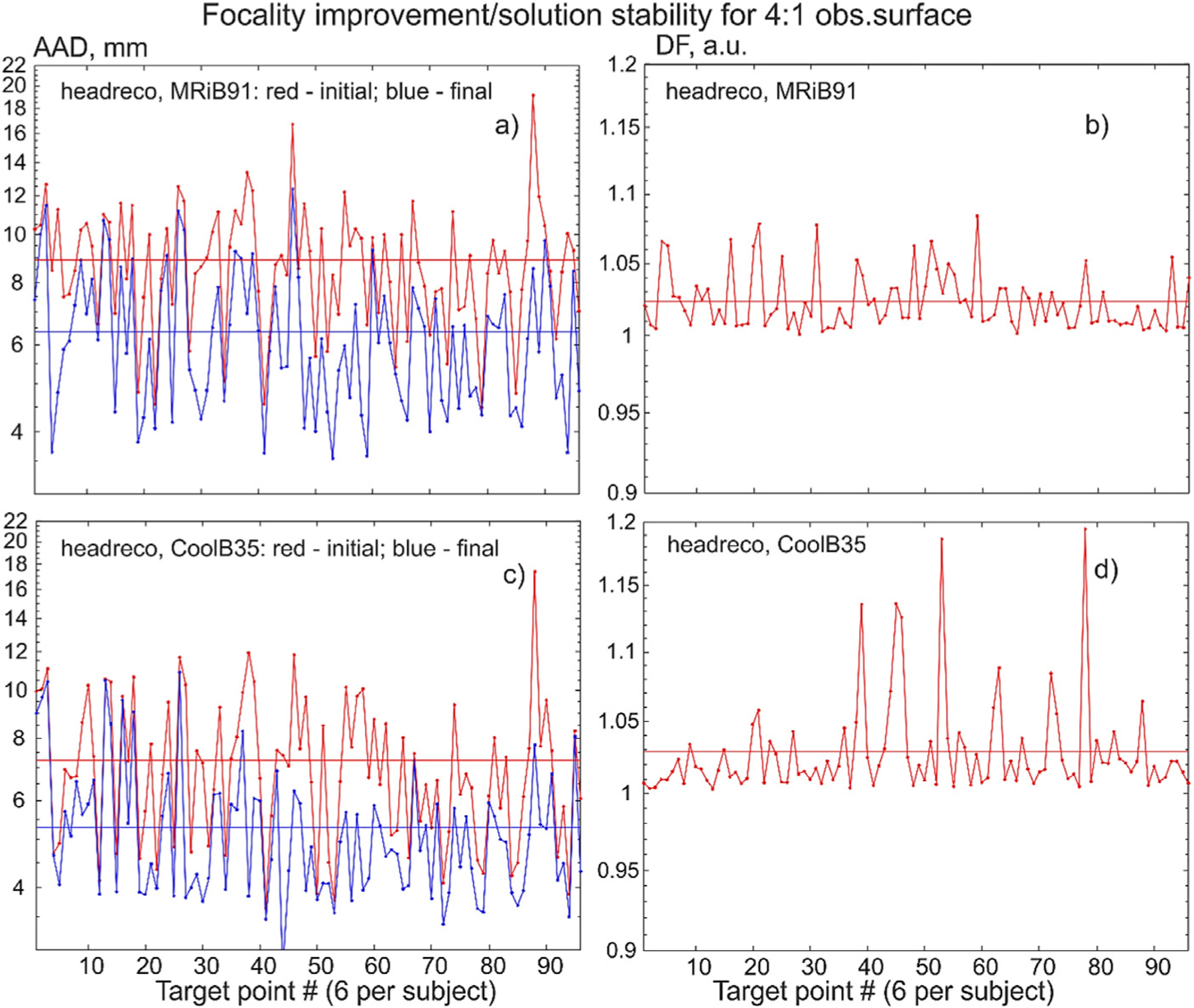
Target-by-target AAD and stability results for the 4:1 surface at the 80% field threshold. Target points are numbered sequentially. Average data are shown by straight lines of the same color.

The corresponding target-by-target individual raw data for all 16 subjects and all target points are illustrated in Fig. 7 below when the cost function with the field threshold of 80% is used. Namely, Fig. 7 shows target-by-target *AAD* in mm and dimensionless defocalization *DF*_*coil*_ results at the 4:1 surface for the headreco segmentation and for the two coil types. The target points are numbered sequentially within each subject model as shown in Fig. 3 so that the argument in Fig. 7 runs from 1 to 96.

### 3.4. Typical field loss for the mid-surface due to TMSP-IP solution

The inverse-problem solution is characterized by an overall field intensity decrease or a field loss at the target point as compared to the sulcus-aligned mapping used as the initial guess. *Four* mid-surface nodes nearest to the target point have been chosen to evaluate the field loss. It is computed in the form

(7) $$Field\;Loss=100\%\times \left(\frac{mean_{4\;neighbors}\left\{E_{\text{sulcus}\;\text{aligned}}\right\}}{mean_{4\;neighbors}\left\{E_{\text{inverse}\;\text{problem}}\right\}}-1\right)$$

**Fig. 8.**
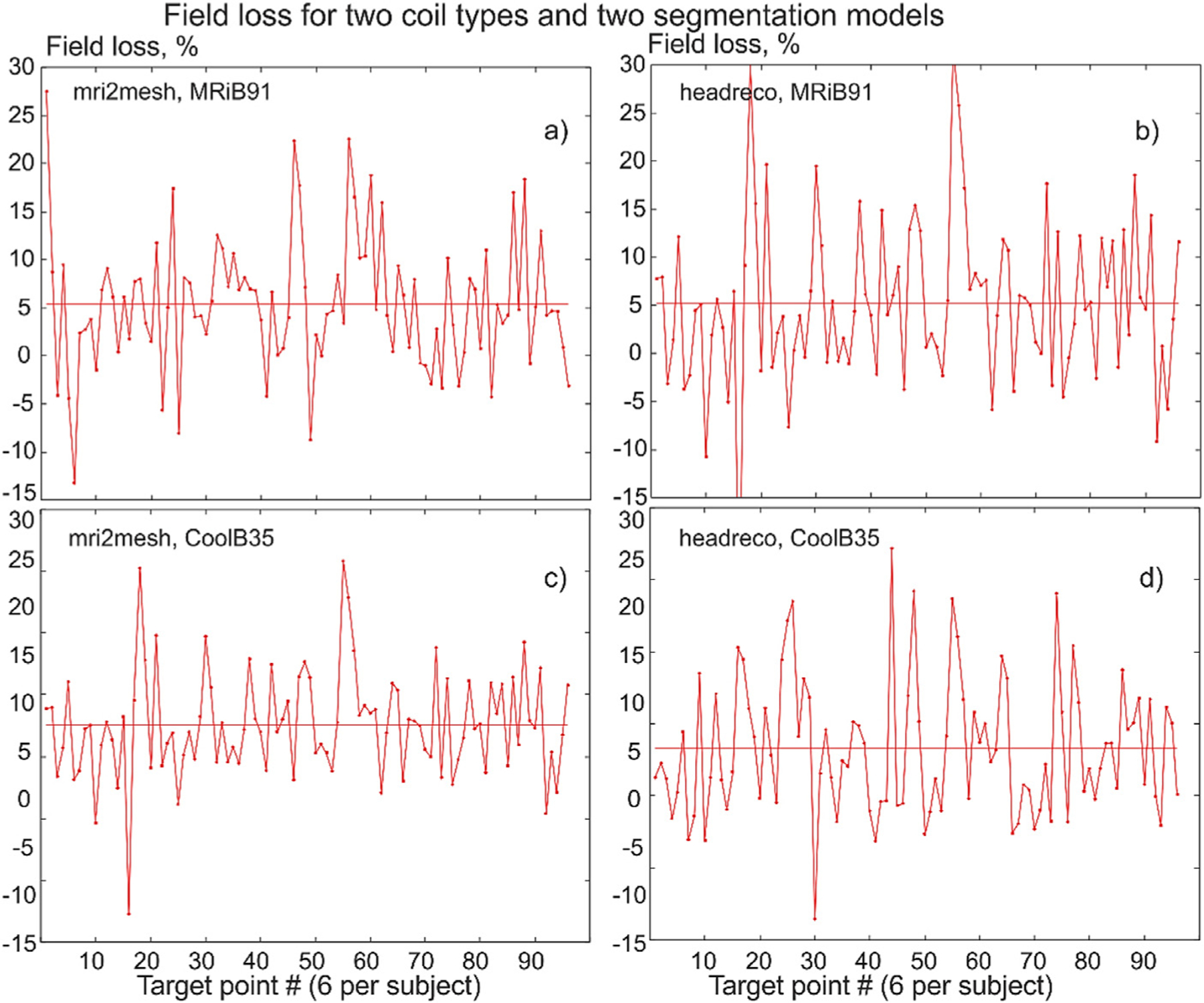
Target-by-target field loss percentage for the mid-surface at the 80% field threshold. The target points are numbered sequentially. Average data are shown by straight lines.

The corresponding target-by target individual raw data are given in Fig. 8 at the 80% field threshold for both segmentation models and for the two coil types. The negative values observed in a number of cases reflect the field gain as compared to the sulcus-aligned mapping. Similar results have been obtained when 10 nearest nodes have been used in Eq. (7).

### 3.5. Effect of segmentation inaccuracy for the mid-surface

**Fig. 9.**
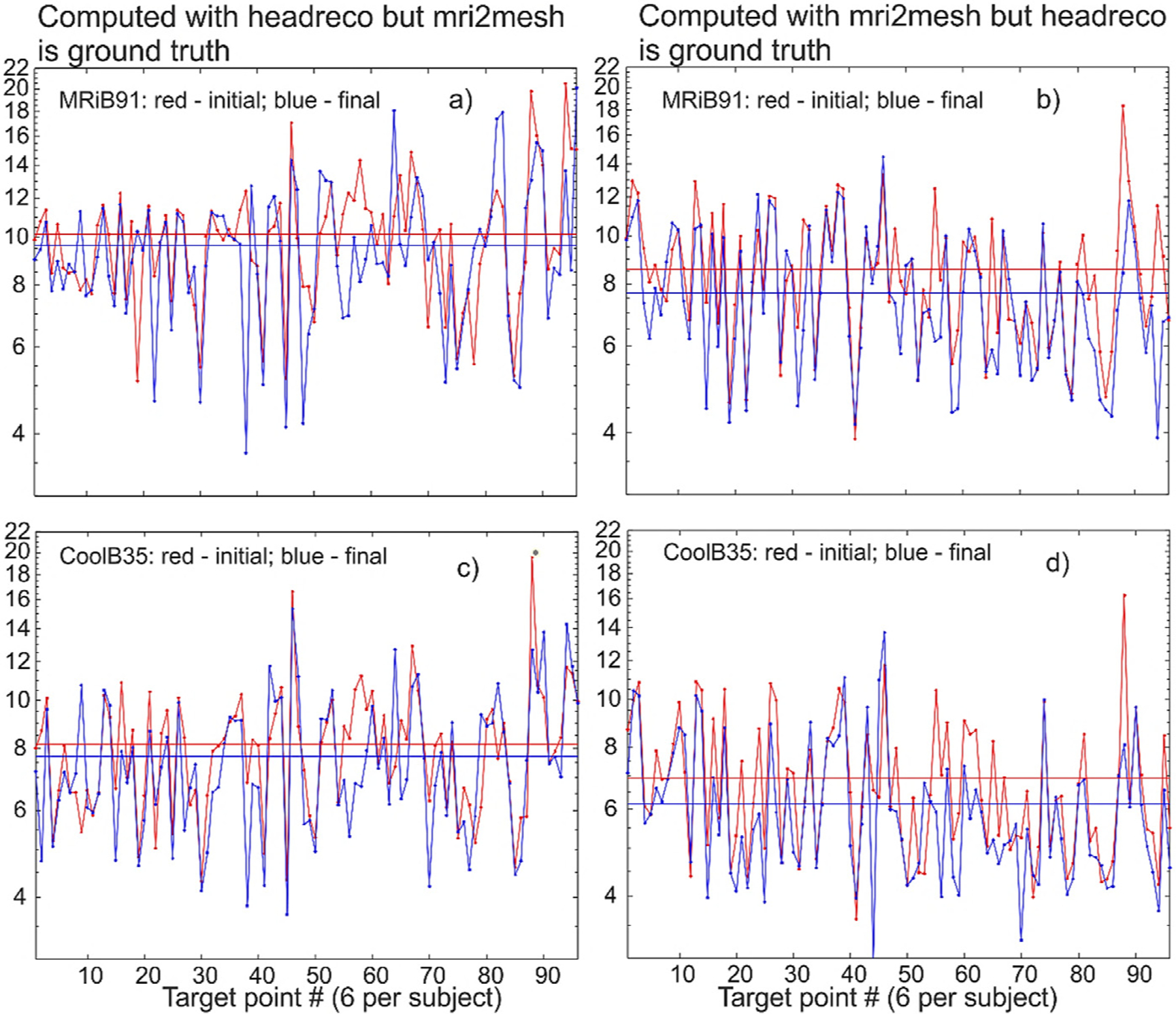
An extreme case illustrating the effect of segmentation uncertainties on the TMS-IP solutions. There is almost no improvement of *AAD* when mri2mesh is the ground truth but optimization is done for headreco in a), c) and a minor improvement when headreco is the ground truth but optimization is done for mri2mesh in b), d). Average data are shown by straight lines of the same color.

Here, two extreme cases will be demonstrated. In the first case, we perform optimization with the headreco segmentation but assume that the ground truth is the mri2mesh segmentation. We therefore compute the final *AAD* for the mri2mesh model but based on the TMS-IP solution for headreco. The results for the two coil types are shown in Fig. 9 a,c, respectively. In the second case, we perform optimization with the mri2mesh segmentation but assume that the ground truth is the headreco segmentation. We therefore compute the final *AAD* for the headreco model but based on the TMS-IP solution for mri2mesh. The results for two coil types are shown in Fig. 9 b,d, respectively Note that the results for the initial *AAD* in Fig. 9 are somewhat different (by 1.5–3%) as compared to Fig. 6 since the normal vectors to the skin surface are slightly different for both segmentations. However, the results for the final *AAD* are *very* different as compared to Fig. 6. A very minor *AAD* improvement is observed in Fig. 9 in general. This is in stark contrast with Fig. 6 and Fig. 7, respectively.

## 4. Averaged results

### 4.1. *Summary of* AAD *focality improvement for the mid-surface*

Table 3 summarizes the average absolute deviation, *AAD*, at the mid-surface before and after the inverse-problem solution, along with the defocalization, *DF*_*coil*_, estimates. Every number in Table 3 is an averaged value for 96 targets points (6 points per subject). The corresponding standard-deviation values are given using a non-bold font.

### 4.2. Reduction in distance to the absolute E-field peak from target after coil optimization

Table 4 reports deviations of the position of the absolute field maximum (computed as the average position of the 99%tile of the field) from the target in mm for the sulcus-aligned mapping and for its TMS-IP improvement, respectively, at the mid-surface and for three different field threshold values. Every number is an averaged value for 96 targets points (6 points per subject for 16 subjects). The standard-deviation (STD) values are given using a small font. Note that the deviation distance for the sulcus-aligned mapping does not depend on the specific field threshold.

### 4.3. *Summary of* ARD *somatotopic resolution improvement and its statistical significance*

It should be noted that the inverse-problem solution is not originally meant to improve the somatotopic differentiation; its original cost function is *AAD*. Nevertheless, a certain improvement (i.e., increase) in *ARD* might also be expected. To prove this, Table 5 summarizes the average relative difference or the somatotopy metric, *ARD*, at the mid-surface before and after the inverse-problem solution, respectively. Data for the two segmentation types and the two coil types are given. STD values are reported using a non-bold font. Statistical significance of the *ARD* improvement versus the sulcus-aligned mapping is quantified via the *p* − *value* for the paired-sample t-test with *p* ≤ 0.05 considered statistically significant.

### 4.4. Summary of field loss for the mid-surface

Table 6 summarizes the field loss or the average ratio of the total field magnitudes before and after the TMS-IP solution, respectively, given by Eq. (7).

### 4.5. *Summary of* AAD *focality improvement for the 4:1 intracortical observation surface between gray and white matter*

The observation surface now approximately corresponds to the bottom of L5 with thick-tufted pyramidal cells with an early bifurcating apical tuft (Aberra et al., 2020). Only the results for the headreco segmentation are shown here at the 80% field threshold. Table 7 summarizes the average absolute deviation, *AAD*, at the 4:1 surface before and after the inverse-problem solution as well as defocalization, *DF*_*coil*_, estimates. Every number is an averaged value for 96 targets points.

### 4.6. Differences in optimized coil positions/orientations for 1:1 and 4:1 intracortical observation surfaces

Table 8 summarizes deviations between the two sets of the TMS-IP results for the final coil position/orientation: one for the 1:1 mid-surface and another for the 4:1 surface. Every number is an averaged value for 96 targets points. The standard-deviation values are given using a non-bold font. Only the results for the headreco segmentation are shown here at the 80% field threshold.

## 5. Location-wise and threshold-wise results

Below, optimization results are presented and visualized per location over subjects. Only results for the headreco segmentation and for the mid-surface are given. For visualization, an average cortical gray matter surface generated using FreeSurfer software Fischl (2012), (FreeSurfer Software Suite 2021) for 40 subjects has been used and the average observation points have been registered against this surface.

### 5.1. *Average* AAD *per target*

**Fig. 10.**
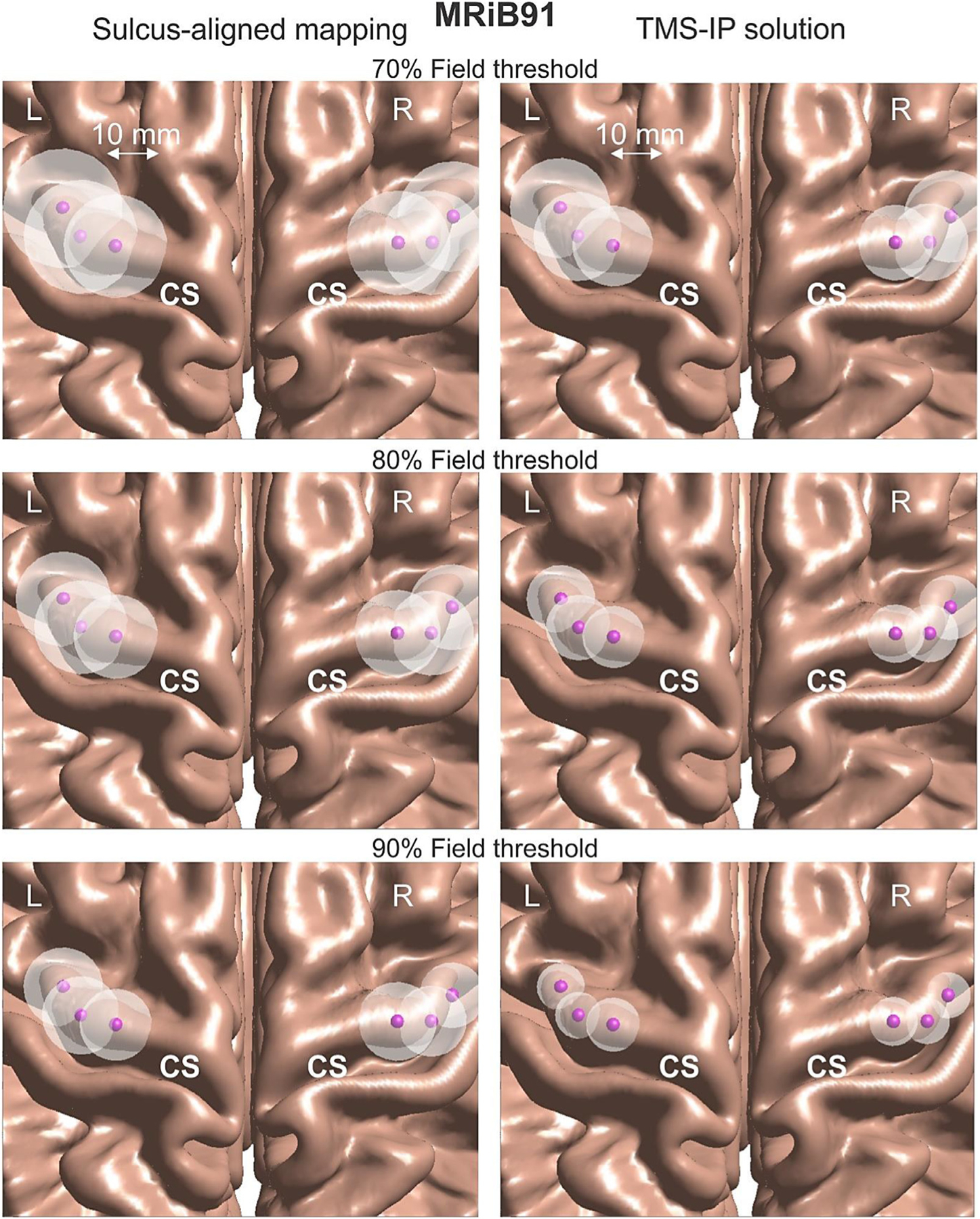
Visualization of average values of *DF*_*cond*_×*DF*_*coil*_×*AAD* for every target location in the M1_HAND_ area for the MRiB91 coil using the average GM surface. Every white sphere centered at the given target has the radius equal to the respective quantity. The left column corresponds to the sulcus-aligned mapping while the right column corresponds to the TMS-IP solution.

**Fig. 11.**
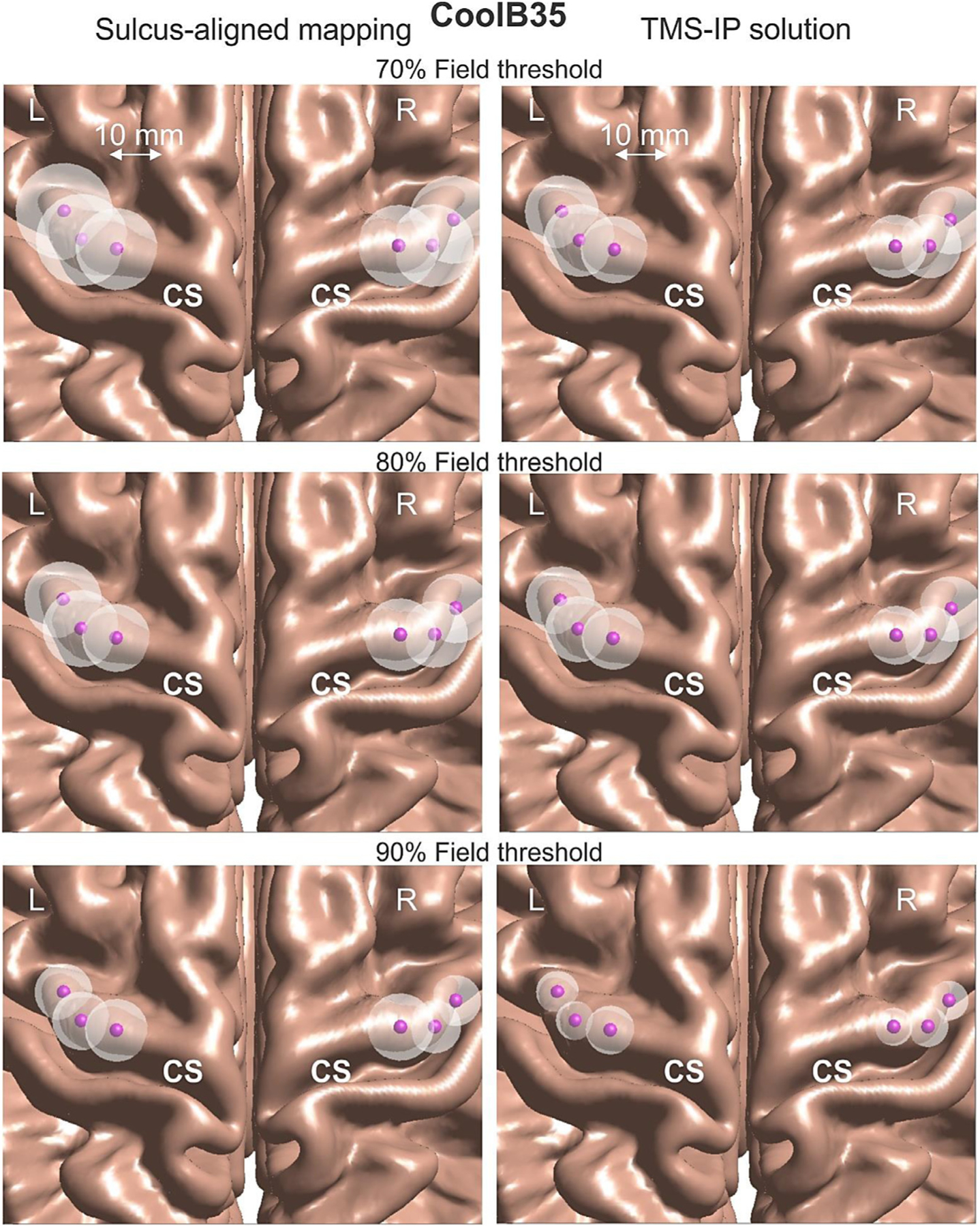
Visualization of average values of *DF*_*cond*_×*DF*_*coil*_×*AAD* for every target location in the M1_HAND_ area for the CoolB35 coil using the average GM surface. Every white sphere centered at the given target has the radius equal to the respective quantity. The left column corresponds to the sulcus-aligned mapping while the right column corresponds to the TMS-IP solution.

The corresponding results are given in Fig. 10 and Fig. 11. In Fig. 10 and Fig. 11, every white sphere centered at the given observation point has the radius equal to the respective *AAD* averaged over 16 subjects and multiplied by the defocalization from Table 3 and by the additional defocalization from Eq. (6) i.e., *DF*_*cond*_×*DF*_*coil*_×*AAD*. The effect of defocalization due to the segmentation uncertainty is not included; it will be discussed separately. Fig. 10 relates to the MRiB91 coil while Fig. 11 describes the results for the CoolB35 coil, respectively, at all three values of the field threshold. For the sulcus-aligned mapping, we assumed the defocalization factor *DF*_*coil*_ equal to that of the TMS-IP solution for the lowest 70% field threshold (1.015 on average).

### 5.2. *Average* ARD *per target – somatotopic resolution maps*

**Fig. 12.**
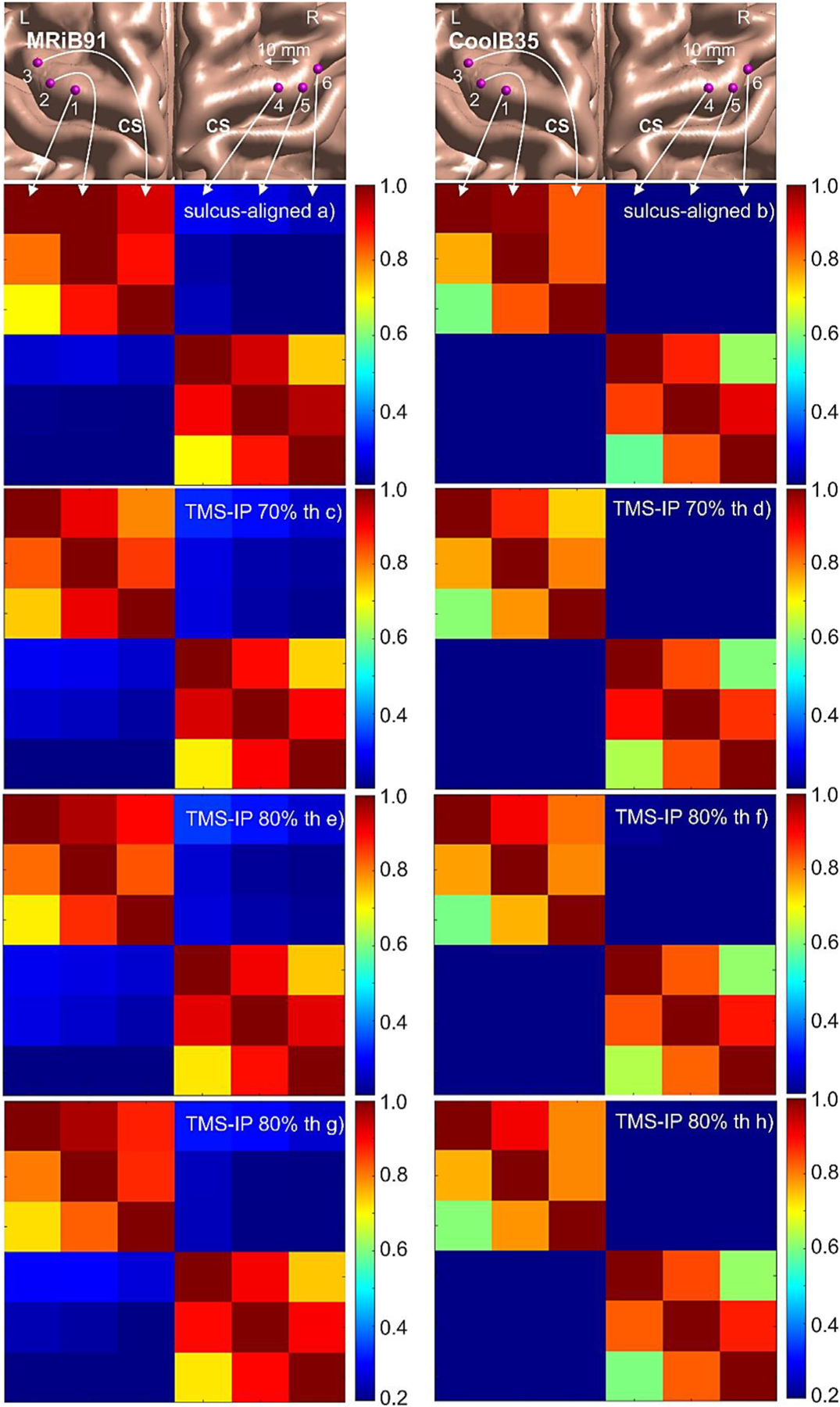
Somatotopic resolution maps – relative field magnitudes at all targets given that one target is excited at a time – a 6 × 6 pixel image of the relative field intensities. a), b) – Maps for the sulcus-aligned mapping for the two coil types. c-h) – Maps for TMS-IP solutions at different values of the field threshold and for the two coil types. Left/right columns are for MRiB91/CoolB35 coils.

Those maps are shown in Fig. 12. Every such map is given by relative field magnitudes at all six targets given that only one target is excited at a time – a 6 × 6 pixel image of the relative field intensities. The normalization is made with respect to the intensity of the excited target. Figs. 12 a,b show the maps for the sulcus-aligned mapping and for the two coil types. These maps do not depend on the field threshold. Figs. 12 c-h present the maps for TMS-IP solutions at different values of the field threshold and for the two coil types.

### 5.3. *Threshold-wise results for* AAD *and* ARD

**Fig. 13.**
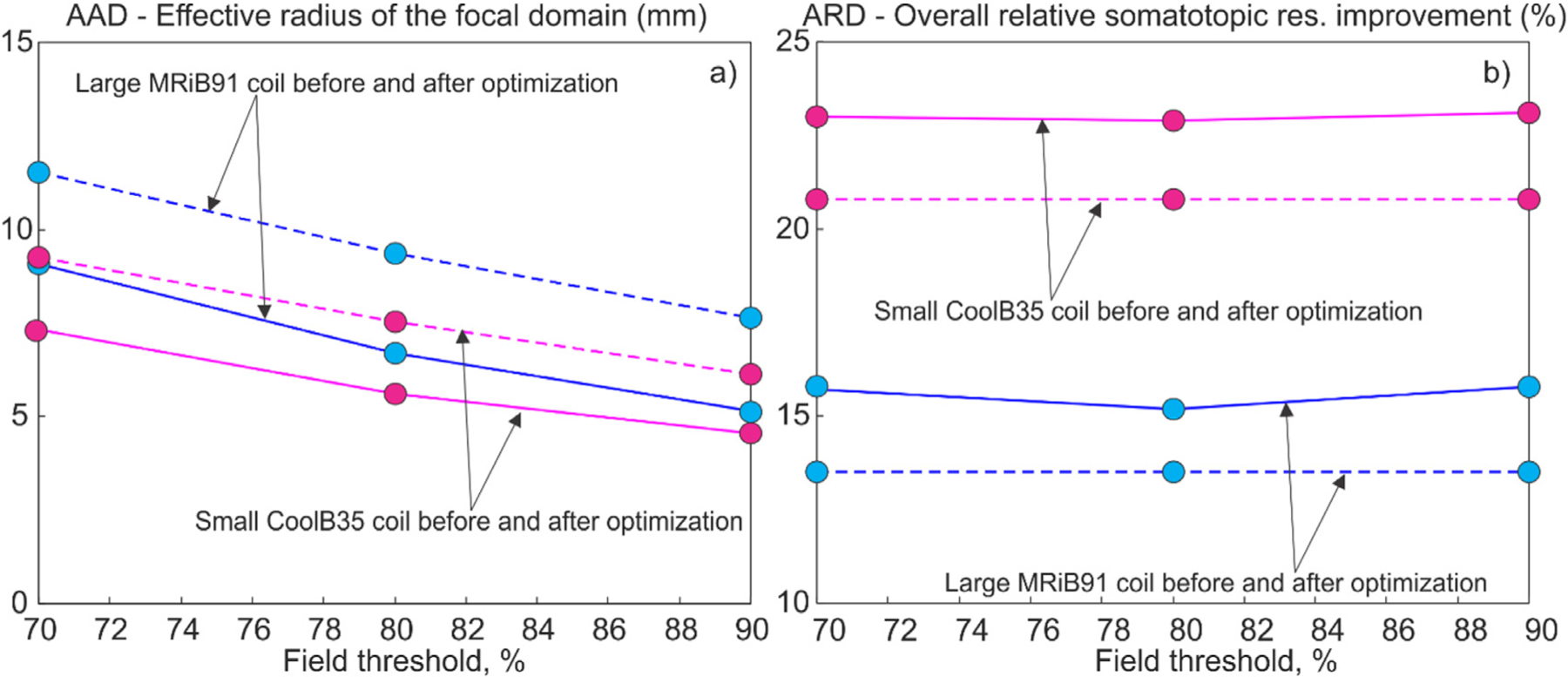
a) – Absolute average *AAD* from Eq. (1) in mm multiplied by the defocalization from Table 3 and by the additional defocalization from Eq. (6), *DF*_*cond*_×*DF*_*coil*_×*AAD*, as a function of the field threshold. The effect of defocalization due to the segmentation uncertainty is not included into consideration. b) – The overall average *ARD* given by Eq 2 as a function of the field threshold. Dashed lines correspond to the sulcus-aligned mapping; solid lines – to the TMS-IP solutions.

Here, average optimization results are presented as a function of the field threshold in the definition of the cost function given by Eq. (1). Only results for the headreco segmentation and for the mid-surface are given. Fig. 13 a illustrates the overall average improvement (or decrease) in *AAD* multiplied by the defocalization from Table 3 and by the additional defocalization from Eq. (6), that is *DF*_*cond*_×*DF*_*coil*_×*AAD*. The effect of defocalization due to the segmentation uncertainty is not included into consideration; it will be discussed separately. Fig. 13 b illustrates the overall average improvement (or increase) in *ARD* given by Eq 2.

## 6. Discussion

In the present study, we perform, arguably for the first time, a rather comprehensive investigation aimed to focus the TMS induced electric field close to a specified target point defined on the gray matter interface in the M1_HAND_ area while otherwise minimizing it. The goal is to numerically evaluate the usefulness and degree of improvement of the TMS-IP (inverse problem) solution relative to the well-known sulcus-aligned mapping. The TMS-IP is an extremely interesting and complicated problem, with many important questions yet to be answered.

Our major finding is that the TMS-IP provides a moderate yet stable average improvement, which implies reducing the size of the “hot spot” of the total electric field and its deviation from the target for the given field threshold. This improvement is consistent with respect to different subjects, different observation points, different coil types, different field observation surfaces (field domains), and different field threshold values used in the cost function.

This hot spot size is determined here via the average absolute deviation (*AAD*) of the field maxima from the target point ***T*** in 3D given by Eq. (1) and evaluated over an observation surface. The observation surface is either the 1:1 mid-surface between the gray and white matter or its 4:1 counterpart located significantly closer to the white matter interface.

Below, we quantify this and other results with respect to different metrics and other conditions while paying special attention to coil angles, attempt to link our modeling results to the recent experimental data (Weise et al., 2020, Raffin et al., 2015), and finally discuss the limitations of the study. Additionally, some results for the white matter interface itself are considered in Appendix A.

### 6.1. *TMS-IP solutions moderately improve* AAD*–based TMS focality by approximately 21-33%*

Based on Tables 3, 7, and Fig. 13, we observe that the TMS-IP solutions provide a moderate improvement of the TMS focality for M1_HAND_ at both the mid-surface (∼L2/3) and the 4:1 (∼L5) surface as compared to the sulcus-aligned motor mapping ($\text{CURVED}-90_{flex}^{\circ }$ (Raffin et al., 2015, Dubbioso et al., 2017)). This improvement given by Eq. (5) – the percentage of the *AAD* reduction as compared to the original *AAD* of the sulcus-aligned mapping – varies from 21% to 33% for the MRiB91 coil and from 21% to 27% for the CoolB35 coil at different values of the field threshold and other parameters. These relative values are reported for the headreco segmentation. They take into account the effect of the coil position/orientation uncertainty while the effect of the conductivity uncertainties cancels out for the relative estimates. The relative improvement increases when the field threshold increases, but it is weakly affected by the coil type. The absolute *AAD* values are indeed smaller for the smaller coil. These results are very consistent.

The corresponding location-wise results are reported in Figs 10 and 11, respectively. No strong variations in the mediolateral direction have been observed in general although some variations could be still seen there when the smaller field thresholds are used.

How do we choose the “optimum” field threshold percentage? Fig. 13 might provide a partial answer. For *AAD* in Fig. 13 and according to Table 3, the field thresholds of 85–90% might still work the best in the present case. Higher values will quickly become unstable: TMS-IP solutions with a 95% field threshold already break down. For *ARD* in Fig. 12 and according to Table 5, any values between 70% and 90% seem to be acceptable although there is also a slight bias toward higher values. These estimates are only valid for the *given* level of uncertainties; they do not take into account the segmentation uncertainty and the expected solution degradation. Thus, the optimum field threshold value should be a function of the expected TMS uncertainties.

### 6.2. TMS-IP solutions for the mid -surface and the 4:1 observation surface nearly coincide

Table 9 presented below gives the percentage of *AAD* reduction as compared to the original *AAD* of the sulcus-aligned mapping for both observation surfaces and at the 80% field threshold.

It summarizes results of Tables 3 and 7. The results for the 1:1 and 4:1 observation surfaces in Table 9 are almost undistinguishable from each other, along with their standard deviations. This observation justifies use of the mid-surface as a good representative for all cortical layers of interest. Additionally, the deviations between the two sets of the TMS-IP results for the coil’s final positions/orientations reported previously in Table 8 are also quite small when the results for both surfaces are compared.

### 6.3. TMS-IP solutions are relatively stable with regard to small variations of coil position and orientation and variations in conductivities

Results reported in Tables 3 and 7 predict an exemplary solution stability at both the 1:1 mid-surface and at the 4:1 observation surface: the average de-focalization (increase in the *AAD* size) does not exceed 2–3% when the coil is moved/rotated by ±1.5 mm and by ±0.1 rad about the optimized position. These dimensions approximately correspond to the accuracy of a robotized TMS navigator or cobot (Axilum Robotics 2021) (controlled 2 mm linear and 4 deg angular coil placement accuracy).

As to the conductivity uncertainties and the relevant defocalization, we employed the results of previous comprehensive relevant studies reported in (Saturnino et al., 2019) and (Weise et al., 2020), respectively, and used the estimate given by Eq. (6) for the corresponding defocalization. The geometrical *AAD* results in Figs. 10 and 11 include both defocalization mechanisms.

### 6.4. TMS-IP solutions are critically affected by segmentation accuracy

Results of Section 3.5 including Fig. 9 indicate that the TMS-IP results are losing their value when the optimization is made with one segmentation (headreco or mri2mesh) but the other segmentation is chosen as the ground truth. The reason is a significant deviation in the brain topology in the M1_HAND_ area illustrated in Fig. 1. A similar observation follows from Table 5. There, one segmentation (mri2mesh) provides no statistically significant improvement of the average relative differentiation (*ARD*) between the targets given by Eq. (2) while the other (headreco) provides a modest yet statistically significant improvement. Tables 3 and 4 also indicate consistently better TMS-IP results for *AAD* and for the deviation of the position of the absolute field maximum from target when headreco segmentation is used, along with the smaller standard deviations. Therefore, we mostly discuss the results for the headreco segmentation considering them more realistic.

### 6.5. TMS-IP solutions consistently reduce distance to the absolute E -field peak from target

Table 4 reports moderate yet consistent reduction in the average distance of the absolute *E*-field peak (99^th^ percentile) from the target point. The distance decreases when the field threshold of the TMS-IP solution increases; it indeed becomes smaller for the smaller coil. The standard deviation values are remarkably low. The smallest average distance approaches 4 mm for the CoolB35 coil at the 90% field threshold and for the headreco segmentation.

### 6.6. TMS-IP solutions improve TMS somatotopic resolution

Although the present inverse-problem solutions are not originally meant to improve the somatotopic differentiation, a certain improvement in *ARD* is observed in Table 5. This improvement is not statistically significant for the mri2mesh segmentation but is becoming statistically significant for the headreco segmentation. There, the *ARD* increases by 2.1% on average in absolute values – see Fig. 13 b – which is a minor improvement as compared to the sulcus-aligned mapping. The relative increase is larger.

However, the location-wise results given in terms of absolute values in Fig. 12 are quite remarkable. With regard to the mediolateral spatial profiles, a substantial (from approximately 0–5% to approximately 20%) average *ARD* improvement is observed for most median targets of M 1 _HAND_ while a smaller improvement is observed for all other targets. Fig. 12 also indicates that the *ARD* metric of Eq. (2) may need a revision since the location-wise results in Fig. 12 demonstrate the improvement in the somatotopic resolution more convincingly and clearly than Table 5.

### 6.7. TMS-IP solutions do not change electric field direction (coil handle direction) significantly

It is well known that the direction of the induced electric field has a large influence on TMS performance (cf. Di Lazzaro et al., 2008, Raffin et al., 2015). Furthermore, TMS of M1_HAND_ excites corticospinal neurons most optimally, if the TMS pulse induces an electrical current that flows perpendicular to the central sulcus in a posterior to anterior direction (Di Lazzaro et al., 2008). Table 10 below shows the deviation of the coil handle angle and its STD between the initial the sulcus-aligned motor mapping ($\text{CURVED}-90_{flex}^{\circ }$ Raffin et al., 2015, Dubbioso et al., 2017) and the final position, respectively, for the 80% field threshold. The mean deviation is rather modest while the maximum deviation never exceeded 45°. All tested deviations of the coil handle angle average to approximately 17°.

### 6.8. TMS-IP solutions slightly lift the coil up to provide space for a small tilt, move it slightly anterior, and do not change the medial-to-lateral position on average. Strictly tangential TMS-IP solutions might yield comparable results

The following numbers characterize average coil movements with respect to the initial sulcus-aligned projection approach given that the field observation surface is the mid-surface. The mean value of the relative vertical coil movement is + 2 mm with the standard deviation of 3 mm. This means that the coil is slightly lifted up to provide some space for a more adequate tilt.

For all considered cases, the coil is moved slightly *anterior* on average, with the mean value of + 1.5 mm and with the standard deviation of 4 mm. The medial-to-lateral movement does not have such an “offset”; it is characterized by an average value that is remarkably close to 0 mm and by the standard deviation of 3 mm. The total coil movement is 6 mm on average with approximately a 4 mm standard deviation for both intracortical observation surfaces (1:1 and 4:1).

A practically interesting question is what happens if we reduce the search space to three parameters i.e., restrict ourselves to the most common tangential coil movements and rotations. The corresponding simulations have been additionally performed for the headreco segmentation and for the mid-surface as the field observation surface. In this case, the improvement reported in Table 9 was reduced from 29% to 20% for the MRiB91 coil and from 26% to 20% for the CoolB35 coil, respectively. In other words, the tangential TMS-IP might be a simpler viable alternative to the more general solution.

### 6.9. TMS-IP solutions require simulation settings adjustment in every individual case

A moderate field loss of approximately 4–6% at target on average (Table 6) is simultaneously observed for all tested configurations after the application of the TMS-IP solution as compared to the sulcus-aligned motor mapping. However, Fig. 8 indicates that these results are very subject/target dependent, with a large standard deviation. Therefore, after optimization, the stimulation settings should have to be adjusted (if possible) using the new *E*-field value at the target if one wants to match the *E*-field in the standard mapping approach (cf., for example, Julkunen et al., 2009, Awiszus, 2003).

If the results for the sulcus-aligned mapping are already available, one possible scenario might be as follows. Assume that the motor threshold and the corresponding stimulator output were already measured for the target and the corresponding *E*-field was also computed. A generic TMS-IP problem with a desired field threshold (e.g., 80% of maximum *E*-field) of the cost function and with the same TMS pulse strength is solved next, which will give us a new value for the target *E*-field due to the potential field loss. The ratio of the two field magnitudes times the desired field threshold of the cost function and multiplied by the initial stimulator output will roughly give us the new stimulator output for the target. Other approaches are certainly possible.

### 6.10. Link to recent experimental data (Weise et al., 2020; Raffin et al., 2015)

In Ref. (Weise et al., 2020), the hotspots (the anticipated target points) of a high congruence factor between the applied electric field and the motor evoked potential (MEP) have been initially determined for three subjects while recording MEPs over the first *dorsal interosseous* muscle belly and at the proximal interphalangeal joint. A CB60 coil of MagVenture was used in that experiment, which is similar in size to the first coil of the present study (MRiB91).

Next, using an exhaustive search with ∼5,000 search points, the coil positions and orientations were found that maximize the electric field magnitude in the hotspots or the target points in M1_HAND_. The subsequently measured (over 16 experiments with the similar target spacing of 7–10 mm) motor thresholds were always lowest for the predicted optimal positions and orientations. This study specifically mentions that these optimal coil orientations were similar to the commonly used 45° angle towards the *fissura longitudinalis* (cf. (Weise et al., 2020 ), Fig. 21) or $\text{CURVED}-45_{fix}^{\circ }$ in terms of Ref. (Raffin et al., 2015).

While we were unable to perform our own relevant experiments, we could establish quantitative correlations with these results with regard to the optimal angular coil positioning. The sulcus-aligned mapping (the initial guess shown for one head model in Fig. 3) gave us the (absolute) angle towards the *fissura longitudinalis* of 29° on average for 16 subjects and 96 target points (cf. Fig. 3). The TMS-IP solution increased this average value to 36° (headreco segmentation) or to 35° (mri2mesh segmentation) for both coil types, with a 19° standard deviation. This means a closer agreement with the observation stated above for the minimum motor threshold angle of 45° on average given the limited angular experimental resolution.

On the other hand, Ref. (Raffin et al., 2015) reports similar experimental somatotopic resolutions for the $\text{CURVED}-90_{flex}^{\circ }$ and $\text{CURVED}-45_{fix}^{\circ }$ approaches, respectively, when *abductor digiti minimi* and *first dorsal interosseus* muscles are concerned. Still, the cross-correlation was the lowest for the $\text{CURVED}-90_{flex}^{\circ }$ approach (Raffin et al., 2015). Our average angular TMS-IP data are almost in between the two approaches too. There is additionally a small yet visible bias toward the $\text{CURVED}-90_{flex}^{\circ }$ approach.

It should be noted that only the average angular directions for the coil handle are reported here. Certain TMS-IP solutions may significantly deviate from them as shown in Figs. 4 and 5, respectively.

### 6.11. Study limitations

The M1_HAND_ area has been the main focus of his study. Along with this, we have also collected and processed some less extensive results related to the dorsolateral left prefrontal cortex (DLPFC), which is pathophysiologically linked to depression (Pascual-Leone et al., 1996, Geller et al., 1997, Koenigs and Grafman, 2009, Kobayashi and Pascual-Leone, 2003). A single observation point was chosen at the middle frontal gyrus of DLPFC, and the inverse problems have been solved for all 16 subjects. Generally, these results look similar to the results reported in this study, but they will need additional verification and extensions to multiple observation points.

As to the numerical modeling, the major drawback of the BEM-FMM approach used in this study is an inability to straightforwardly include into consideration the white matter anisotropy, which may be quite significant in the subcortical white matter (Katoch et al., 2019) and which may have a substantial effect of the *E*-field distribution (Saturnino et al., 2019). On the other hand, this approach is free from the volumetric field averaging throughout the gray matter volume and from the fictitious volumetric charges generated by the first-order FEM. The method requires a relatively small amount of RAM (6–12 Gbytes for the present head models), but it runs best on multicore machines due to inherent FMM parallelization.

Powerful mathematical tools have recently been developed and implemented (Gomez et al., 2021, Daneshzand et al., 2021) for fast computations of the TMS-IP solutions via the auxiliary dipole method (ADM) or the magnetic stimulation profile approach, for determining the optimum coil position and orientation. The goal of the present study is not to compete with these tools but rather to evaluate the usefulness and degree of improvement of the TMS-IP solution itself. We attempted to answer the question by how much the TMS-IP solution can improve the focality of one common projection approach – the $\text{CURVED}-90_{flex}^{\circ }$ mapping –and by how much it can help us to better differentiate between the neighbor targets. For this purpose, we employ a much slower but more general traditional approach. This approach might be more flexible for studying multiple geometrical model variations and arbitrary field observation surfaces, arbitrary coil positions (slightly) different from those strictly tangential to the scalp, and it does not require extra mesh conditioning.

From the practical point of view, the solution of a particular TMS-IP will likely be best accomplished by using specialized highly efficient algorithms such as (Gomez et al., 2021, Daneshzand et al., 2021) instead of the straightforward yet slow approach of this study.

## 7. Conclusions

The TMS inverse-problem solutions studied in this paper predict the stable focality improvement of approximately 21–33% on average for the total electric field in the M1_HAND_ area with its maximum located mostly close to the gyral crown. This estimate is valid for all considered subjects and target points, for two distinct coil types, for both intracortical observation surfaces under study, and for three different values of the field threshold used in the cost function. The solution is using the average absolute deviation, *AAD*, from target given by Eq. (1) as the cost function. The predicted improvement is relative to the projection-based perpendicular-to-sulcus initial coil placement or the sulcus-aligned mapping ($\text{CURVED}-90_{flex}^{\circ }$ Raffin et al., 2015, Dubbioso et al., 2017) which appears to be an excellent initial guess. Along with this, the TMS-IP solutions do not significantly change the electric field direction (the coil handle direction) and lead to a moderate field loss of approximately 4–6% on average.

The inverse-problems solutions simultaneously improve the somatotopic (target-to-target) TMS resolution although this improvement is quite modest on average. At the same time, the corresponding improvement may be significant for most median targets of the M1_HAND_ area. Additionally, the TMS-IP solutions consistently reduce distance to the absolute *E*-field peak from the target.

Coil position/orientation and conductivity uncertainties have been included into consideration as the corresponding defocalization factors. The present results will change when the levels of uncertainties change. The results could be expanded by considering more representative cases and by performing a more rigorous uncertainty power analysis (Weise et al., 2020).

The TMS-IP solutions strongly depend on the segmentation accuracy. For the extreme case when the solution for one realistic segmentation model (mri2mesh or headreco) is used in another model and vice versa, the TMS-IP solution would generate a minor, if any, improvement. Nearly all TMS-IP results for the headreco segmentation are substantially better than the results for the mri2mesh segmentation.

The mention of commercial products, their sources, or their use in connection with material reported herein is not to be construed as either an actual or implied endorsement of such products by the Department of Health and Human Services, USA.

## Figures and Tables

**Table 1:** Subjects, models, and methods of the TMS inverse-problem solution.

Type	Number	Short description
Subjects	**16**	Connectome (Van Essen et al., 2012) subjects 101309, 110411, 117122, 120111, 122317, 122620, 124422, 128632, 130013, 131722, 138534, 149337, 149539, 151627, 160123, 198451
Brain segmentation models (surfaces)	**2**	mri2mesh (based on FreeSurfer (Fischl, 2012) and FSL) or headreco (based on SPM/CAT (Structural Brain Mapping Group 2021)), both from SimNIBS.
Coil models	**2**	Small CoolB35 coil (Fig. 2 a) and a relatively large MRiB91 coil (Fig. 2 b), both of MagVenture.
Target domains	**1**(**l+r**)	M1_HAND_ of the left hemisphere, M1_HAND_ of the right hemisphere.
Target points per domain	**3+3** (**l+r**)	Three target points separated by approx.10 mm on gray matter interface within each M1_HAND_ domain (Fig. 3).
Initial guess	**1**	Sulcus-aligned mapping: 90°_flex_ (Raffin et al., 2015, Dubbioso et al., 2017) as an initial guess
Field observation domain	**1**	Mid-surface between gray/white matter (1:1, ∼Layer 2/3)
Field type	**1**	Total field magnitude
Cost function (focality metric)	**1**	Average absolute deviation (AAD) of the field from target – Eq. (1) below
Field threshold vs maximum field to estimate size of the field “hot spot”	**3**	70%, 80%, 90% (interpolation is possible)
Somatotopy improvement metric	**1**	Average relative difference (ARD) in the field between neighboring target points – Eq. (2) below
Search method	**1**	Gradient descent in the **ℜ**^6^ search space (coil pos. + orient.)
Forward solver	**1**	BEM-FMM (Makarov et al., 2018, Makarov et al., 2020)
Uncertainty sources under study	**2**	Coil position/orientation uncertainties, conductivity and segmentation model uncertainties

**Table 2:** Average distance, *d*, in millimeters between the CSF shells, the gray matter (GM) shells, and the white matter (WM) shells for both segmentation routines used in this study. Subject numbers (first row of the table) correspond to the Connectome list from Table 1.

#	1	2	3	4	5	6	7	8	9	10	11	12	13	14	15	16
*d_CSF_*	0.9	1.5	0.8	0.7	0.8	0.7	0.7	0.9	0.7	1.0	0.7	0.8	1.1	0.8	0.7	1.2
*d_GM_*	0.4	0.3	0.3	0.3	0.4	0.4	0.4	0.4	0.3	0.4	0.3	0.4	0.3	0.4	0.3	0.4
*d_WM_*	0.3	0.3	0.2	0.3	0.2	0.2	0.2	0.3	0.2	0.4	0.2	0.3	0.2	0.2	0.3	0.3

**Table 3:** Top: field focality *AAD* – the effective radius of the focal domain in mm – for sulcus aligned mapping and for its TMS-IP improvement, respectively, at the mid-surface and for three different field threshold values. Bottom: respective relative de-focalization (a.u.) due to coil position/orientation uncertainty. Every number is an averaged value for 96 targets points (6 points per subject for 16 subjects). The standard-deviation (STD) values are given using a non-bold font.

Segmentation type	mri2mesh		headreco	
Coil type	MRiB91	CoolB35	MRiB91	CoolB35
*AAD* in mm for sulcus-aligned mapping (7*0% field threshold*)	**12.10** 2.5	**9.79** 2.1	**10.89** 2.1	**8.71** 1.9
*AAD* in mm optimized via TMS-IP solution (7*0% field threshold*)	**9.91** 2.1	**7.97** 1.7	**8.56** 2.1	**6.83** 1.7
*AAD* in mm for sulcus-aligned mapping (8*0% field threshold*)	**9.99** 2.8	**7.94** 2.4	**8.81** 2.5	**7.12** 2.1
*AAD* in mm optimized via TMS-IP solution (8*0% field threshold*)	**7.67** 2.2	**5.86** 1.5	**6.27** 2.3	**5.25** 1.7
*AAD* in mm for sulcus-aligned mapping (9*0% field threshold*)	**8.61** 3.9	**6.38** 3.0	**7.21** 3.4	**5.80** 2.9
*AAD* in mm optimized via TMS-IP solution (9*0% field threshold*)	**5.58** 2.9	**4.62** 2.4	**4.71** 2.1	**4.13** 1.8
Defocalization, *DF_coil_*, a.u. (7*0% field threshold*)	**1.01**	**1.01**	**1.01**	**1.02**
Defocalization, *DF_coil_*, a.u. (8*0% field threshold*)	**1.02**	**1.03**	**1.02**	**1.02**
Defocalization, *DF_coil_*, a.u. (9*0% field threshold*)	**1.05**	**1.03**	**1.04**	**1.05**

**Table 4:** Deviation of the position of the absolute field maximum (computed as the average position of the 99%tile of the field) from the target in mm for sulcus-aligned mapping and for its TMS-IP improvement, respectively, at the mid-surface and for three different field threshold values. Every number is an averaged value for 96 targets points (6 points per subject for 16 subjects). The standard-deviation (STD) values are given using a non-bold font. Note that the deviation distance for the sulcus-aligned mapping does *not* depend on the field threshold.

Segmentation type	mri2mesh		headreco	
Coil type	MRiB91	CoolB35	MRiB91	CoolB35
Deviation in mm for sulcus-aligned mapping (7*0,80,90% field threshold*)	**8.7** 5.6	**5.7** 4.1	**7.0** 4.9	**5.3** 3.5
Deviation in mm optimized via TMS-IP solution (7*0% field threshold*)	**8.3** 4.8	**6.0** 4.0	**6.5** 4.6	**5.4** 3.3
Deviation in mm optimized via TMS-IP solution (8*0% field threshold*)	**7.9** 4.9	**5.1** 3.4	**6.1** 4.1	**4.7** 2.9
Deviation in mm optimized via TMS-IP solution (9*0% field threshold*)	**6.5** 4.9	**4.6** 3.3	**4.9** 3.4	**4.0** 2.3

**Table 5:** *ARD* percentage and its improvement at the mid-surface for three different values of the field threshold. Every number is an averaged value for 96 targets points (6 points per subject for 16 subjects). STD values and *p* − *values* are given using a small font. Note that *ARD* for sulcus-aligned mapping does *not* depend on the field threshold. Very similar results have been obtained for the 4:1 observation surface at the 80% field threshold.

Segmentation type	mri2mesh		headreco	
Coil type	MRiB91	CoolB35	MRiB91	CoolB35
*ARD*, % for sulcus-aligned mapping (7*0/80/90% field threshold*)	**14.2** 14.4	**21.4** 15.5	**13.5** 20.0	**20.8** 21.1
*ARD*, % for inverse-problem solution (7*0% field threshold*)	**14.6** 13.9 ***p*-value = 0.64**	**22.4** 14.9 ***p*-value = 0.67**	**15.7** 16.1 ***p*-value = 0.07**	**23.0** 17.8 ***p*-value = 0.03**
*ARD*, % for inverse-problem solution (8*0% field threshold*)	**14.8** 14.0 ***p*-value = 0.43**	**22.3** 14.2 ***p*-value = 0.24**	**15.2** 20.2 ***p*-value = 0.05**	**22.9** 21.5 *p***-value = 0.01**
*ARD*, % for inverse-problem solution (9*0% field threshold*)	**15.1** 12.7 ***p*-value = 0.22**	**22.4** 15.5 ***p*-value = 0.11**	**15.8** 18.6 ***p*-value = 0.008**	**23.1** 19.3 ***p*-value = 0.002**

**Table 6:** Field loss for four mid-surface nodes nearest to the target after optimization at three different values of the field threshold. Every number is an averaged value for 96 targets points (6 points per subject for 16 subjects). STD values are given using a non-bold font. Very similar results have been obtained for the 4:1 observation surface at the 80% field threshold.

Segmentation type	mri2mesh		headreco	
Coil type	MRiB91	CoolB35	MRiB91	CoolB35
Field loss, % (7*0% field threshold*)	**4.8/**7.3	**5.7/**9.5	**4.7**/8.7	**5.9/**11.5
Field loss, % (8*0% field threshold*)	**5.4/**6.9	**6.2/**8.7	**5.2**/8.5	**6.6/**9.4
Field loss, % (9*0% field threshold*)	**5.2/**6.7	**5.2/**9.1	**4.1**/7.6	**4.0/**9.1

**Table 7:** Focality improvement and uncertainty driven defocalization at the 4:1 observation surface and at the 80% field threshold. Every number is an average for 96 targets points. STD values are given using a small font.

Coil type	MRiB91	CoolB35
*AAD* for sulcus-aligned mapping, mm	**8.91/**2.8	**7.22**/2.4
*AAD* optimized via TMs-IP, mm	**6.37/**2.2	**5.31**/1.5
Defocalization, *DF_coil_*, a.u.	**1.02**	**1.03**

**Table 8:** Deviations between the two sets of the TMS-IP results for the final coil position/orientation at the 80% field threshold: the 1:1 mid-surface optimization versus the 4:1 surface optimization results. Every number is an average for 96 targets points. Standard-deviation values are given using a non-bold font.

Coil position/angle deviation	MRiB91	CoolB35
Average medial-lateral deviation (*x*), mm	**0.0/**2.4	**−0.1**/2.4
Average posterior-anterior deviation (*y*), mm	**0.0/**3.5	**−0.1**/2.7
Average superior-inferior deviation (z), mm	**0.3/**2.7	**0.0/**2.2
Deviation in coil handle (*E*-field) angle, deg	**0.4/**9.8	**0.4/**10.3

**Table 9:** Percentage of the *AAD* reduction as compared to the original *AAD* of the sulcus-aligned mapping at the 80% field threshold. Every number is an averaged value for 96 targets points (6 points per subject for 16 subjects). The standard-deviation values are given using a small font.

Segmentation type	mri2mesh		headreco	
Coil type	MRiB91	CoolB35	MRiB91	CoolB35
*AAD* reduction for L2/3 mid-surface, %	**23** 14	**26** 15	**29** 17	**26** 16
*AAD* reduction for 4:1 L5 surface, %	**24** 14	**27** 16	**29** 17	**26** 15

**Table 10:** Deviation of the coil handle angle and its STD between the initial sulcus-aligned motor mapping ($\text{CURVED}-90_{flex}^{\circ }$ Dubbioso et al., 2017) position and the final position, respectively, for the 80% field threshold. Every number is an averaged value for 96 targets points (6 points per subject for 16 subjects). The standard-deviation values are given using a non-bold font.

Segmentation type	mri2mesh		headreco	
Coil type	MRiB91	CoolB35	MRiB91	CoolB35
Mean angle deviation, deg	**16** 11	**17** 12	**18** 12	**17** 11

**Fig. A1.**
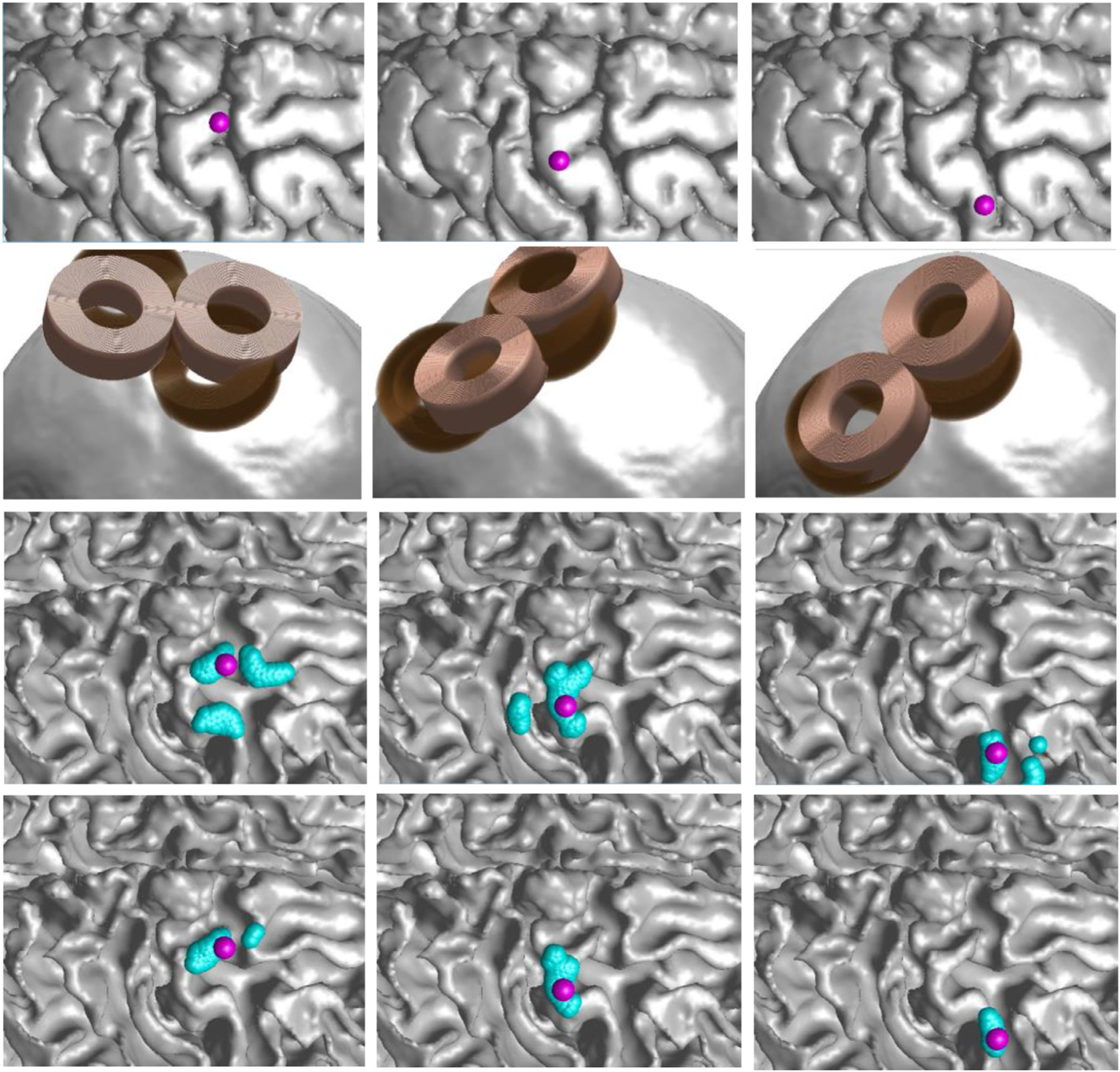
First row – target points in the primary motor cortex for subject #101309. Second row – initial (dark copper) and final (light copper) coil positions for the three optimization cases. Third row – total field for the initial guess just outside the white matter interface in the range of 80–100% of the maximum field or the 80^th^ percentile. Fourth row – the same result after TMS-IP solution (fourth row). The target points are again shown.

## Appendix A TMS-IP solution for white matter observation surface

The total field on the white matter interface of a segmentation model is discontinuous due to the conductivity discontinuity. It can be computed just inside or just outside the interface (Makarov et al., 2020). This is, strictly speaking, a non-physical effect: the myelin concentration increases gradually when passing through the interface. However, the corresponding transition domain is relatively thin and the gray-white differentiation can be done on a submillimeter scale (cf., for example, Stüber et al., 2014).

### A1. TMS-IP solution for the total field in the primary motor cortex

As far as the total field is concerned, the results for the optimization just inside and outside the white matter interface appear similar but not identical. Table A1 present results for the total field optimization just outside the white matter interface. In this case, an approximate center of the M1_HAND_ area of the right hemisphere has been selected for every subject as a target point, and the results were then averaged for all 16 subjects.

This table simultaneously presents the results for the normal field optimization, for the CoolB35 coil. The normal fields just inside and outside correlate with each other (Makarov et al., 2020) and are optimized identically. These results appear to be quite different from the total-field results (a better optimization and a higher instability). Note that the normal field at the white matter interface may dominate the tangential field.

To highlight the behaviour of the field optimization on the white matter interface, Fig. A1 illustrates the optimization results for several target points in the primary motor cortex area of subject #101309. The total field just outside the white matter interface is optimized. The initial target points on the gray matter interface are shown by magenta spheres in the first row.

**Table A1:** Focality and its improvement, field loss, and the defocalization due to coil position/orientation uncertainties for the white matter surface optimization (just outside) at the 80% field threshold. Every number is an averaged value for 16 subjects. For the CoolB35 coil, results for total and normal fields are given simultaneously.

Coil type	MRiB91	CoolB35	
Field type	Total	Total	Normal
*AAD* for sulcus-aligned mapping, mm	**12.0**	**8.8**	**11.5**
*AAD* optimized via TMS-IP, mm	**8.1**	**5.9**	**6.4**
Defocalization, *DF*_*coil*_, a.u.	**1.02**	**1.03**	**1.21**
Avg. field loss, %	**12**	**15**	**17**
Avg. coil movement, mm	**8.5**	**7.8**	**10.0**
